# Impacts of resistant starch and wheat bran consumption on enteric inflammation in relation to colonic bacterial community structures and short-chain fatty acid concentrations in mice

**DOI:** 10.1186/s13099-016-0149-6

**Published:** 2016-12-22

**Authors:** Janelle A. Jiminez, Trina C. Uwiera, D. Wade Abbott, Richard R. E. Uwiera, G. Douglas Inglis

**Affiliations:** 1 Agriculture and Agri-Food Canada, 5403-1st Avenue South, Lethbridge, AB T1J 4B1 Canada; 2 Department of Agricultural Food and Nutritional Science, University of Alberta, 410 Agriculture/Forestry Centre, Edmonton, AB T6G 2P5 Canada; 3 Divisions of Pediatric Surgery, Department of Surgery, University of Alberta, 2C3.82 Walter C. Mackenzie Health Sciences Center, 8440-112th Street, Edmonton, AB T6G 2B7 Canada

**Keywords:** Resistant starch, Wheat bran, *Citrobacter rodentium*, Acute enteritis, Inflammation, Short-chain fatty acids

## Abstract

**Background:**

Identifying the connection among diet, the intestinal microbiome, and host health is currently an area of intensive research, but the potential of dietary fiber (DF) consumption to ameliorate intestinal inflammation has not been extensively studied. We examined the impacts of the DFs, wheat bran (WB) and resistant starch (RS) on host enteric health. A murine model of acute Th1/Th17 colitis (i.e. incited by *Citrobacter rodentium*) was used.

**Results:**

Diets enriched with RS increased weight gain in mice inoculated with *C. rodentium* compared to mice consuming a conventional control (CN) diet. Short-chain fatty acid (SCFA) quantities in the cecum and distal colon were higher in mice consuming DFs, and these mice exhibited higher butyrate concentrations in the distal colon during inflammation. Histopathologic scores of inflammation in the proximal colon on day 14 post-inoculation (p.i.) (peak infection) and 21 p.i. (late infection) were lower in mice consuming DF-enriched diets compared to the CN diet. Consumption of WB reduced the expression of Th1/Th17 cytokines. As well, the expression of bacterial recognition and response genes such as *Relmβ*, *RegIIIγ*, and *Tlr4* increased in mice consuming the RS-enriched diets. Furthermore, each diet generated a region-specific bacterial community, suggesting a link between selection for specific bacterial communities, SCFA concentrations, and inflammation in the murine colon.

**Conclusions:**

Collectively, data indicated that the consumption of DF-rich diets ameliorates the effects of *C. rodentium*-induced enteritis by modifying the host microbiota to increase SCFA production, and bacterial recognition and response mechanisms to promote host health.

**Electronic supplementary material:**

The online version of this article (doi:10.1186/s13099-016-0149-6) contains supplementary material, which is available to authorized users.

## Background

The intestine is highly influential to host health and contributes greatly to the balance and regulation of the host intestinal immune system and the microbiome. The association between the quality of foods consumed and the severity of intestinal diseases has become a topic of scientific interest, and diets rich in dietary fibers (DFs) are suggested to reduce intestinal inflammation [1, 2]. Dietary fibers are defined as carbohydrates with three or more polymerized saccharide units that resist digestion in the small intestine by host-derived intestinal enzymes, and are only susceptible to fermentation by bacteria in the intestine [3]. They differ from one another in their water solubility, viscosity, and effects on the intestinal microbiota [4, 5]. Fermentation rates can vary depending on the chemical structure of DFs, and characteristics such as chain length, quantity of saccharide units, and the number of sugar linkages regulate the efficiency of carbohydrate fermentation [6]. Certain enteric bacterial species possess specialized metabolic enzymes that ferment specific forms of DFs, and products of fermentation contribute to the bacterial diversity within the intestinal microbiota [4, 7, 8]. By-products of bacterial fermentation are mainly composed of short-chain fatty acids (SCFAs) such as acetate, propionate, and butyrate [9, 10] as well as gases. Acetate and propionate are primarily metabolized in the liver and are important for gluconeogenesis and liponeogenesis [11]. In contrast, butyrate is a primary energy source for colonocytes, and it is also thought to enhance mucus production and regulate intestinal immune function [12]. Other putative functions of SCFAs within the intestine include maintaining homeostatic intestinal pH and mucosal osmolarity [10], and influencing mucin production and secretion [13], all of which are considered important for both cellular and microbial functions.

Resistant starches (RSs) are highly soluble and readily fermented fibers that produce high quantities of butyrate following bacterial fermentation [14]. Resistant starch is primarily comprised of amylose and branched-amylopectin carbohydrate units, and exist as many complex structures that differ in their accessibility to enzymatic digestion [9]. The digestion of RS by intestinal bacteria can affect the structure of the microbiota within the large intestine, and RS fermentation often leads to an increase in *Bifidobacterium* spp., *Parabacteroides* spp., *Ruminococcus* spp., and *Eubacterium* spp. [15, 16]. The microbial fermentation of RS increases intestinal SCFA concentrations, which lower the intestinal pH to modulate bacterial growth, and increase water and ion uptake to counter the losses experienced during diarrheic events [17]. Furthermore, the fermentation of high amylose starch has been associated with influencing muscular contractions in the large intestine that increases the blood flow in viscera and counters the negative effects of diarrhea [17]. Wheat bran (WB) is a DF that is not as easily fermented when compared to RS, but its fermentation can lead to an increase in overall SCFA production in the intestine [18]. Wheat bran is a complex molecule composed mainly of insoluble non-starch polysaccharides, including arabinoxylan, cellulose, and β-glucan fractions that comprise ≈46% of its total fiber content [5, 19]. The complex structure and low percentage of soluble fiber present in WB contributes to slower fermentation rates by intestinal bacteria, and as such, is important for the increase in fecal bulking and stool frequency [20]. The fermentation of WB also improves intestinal lipid profiles that may lower the risk of intestinal cancer development [4, 7, 21]. Similarly to RS digestion, bacterial fermentation of WB can affect bacterial communities within the intestine. For example, fermentation of WB increases the abundance of *Clostridia* cluster XIVa, including butyrogenic bacteria such as *Faecalibacterium prausnitzii* and *Roseburia intestinalis* [22]. Although RS and WB both alter the intestinal microbiota and affect intestinal function by increasing water uptake and fecal bulking respectively, these DFs appear to influence the intestinal environment and immune response in different ways. Thus, we chose to examine the impacts of both RS and WB on the enteric microbiota and host to elucidate key aspects of the relationship among diet, host responses, and inflammation.

*Citrobacter rodentium* is a Gram negative bacterium that incites epithelial cell hyperplasia and inflammation within the murine colonic mucosa, and it is commonly used to study intestinal inflammation in mice [23]. Colitis incited by *C. rodentium* is a self-limiting process in mice, with peak infection occurring ≈7–14 days post-inoculation (p.i.) and disease resolution occurring ≈28 days p.i. [24], although disease can be prolonged up to 3–4 weeks p.i. [25]. *C. rodentium* induced tissue injury is associated with transmural inflammatory changes, and once the bacterium breaches the mucosa it subsequently invades the underlying tissue [26–28]. Infections with *C. rodentium* induce a pro-inflammatory immune response that is characterized by an initial Th17 response, followed by a Th1 response towards recovery [27, 29]. This bacterium is commonly used as an inducer of acute inflammation in the murine intestine [23], but few studies have examined the impacts of DFs on the pathobiology of *C. rodentium*-induced intestinal inflammation. Generally, research investigating the therapeutic potential of DFs has focused on the alterations to the microbiota in models that do not exhibit acute inflammation [30–32]. A number of studies have investigated the effects of DFs on the host using inflamed enteric models. However, these studies have primarily induced inflammation using chemical incitants [33–37], and very few studies have used a bacterial incitant to induce intestinal dysbiosis [38]. In the current study, we examined the effects of the DFs, RS and WB collectively on the intestinal microbiota and the host, with an emphasis on the host immune response following challenge with *C. rodentium* as a Th1/Th17 inflammation incitant. We hypothesized that DFs will increase bacterial fermentation and SCFA concentrations within the large intestine of mice. This will stimulate enhanced mucus secretion, alter luminal bacterial communities including selection for butyrogenic taxa, and modulate the intestinal immune response to ameliorate intestinal inflammation. We further hypothesized that as RS will be more readily fermented than WB, the fermentation of RS will contribute to an increased amount of intestinal butyrate, and thereby differentially increase mucin secretion to reduce *C. rodentium*-induced mucosal injury.

## Results

### *Citrobacter rodentium* incited enteritis

**Fig. 1:**
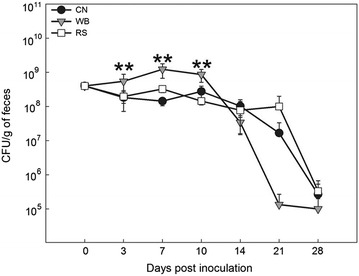
Densities of *Citrobacter rodentium* (CFU/g) in feces from mice inoculated with the bacterium over a 28 day period consuming a control diet (CN), or a diet enriched with wheat bran (WB) or resistant starch (RS). *Vertical lines* associated with *markers* represent the standard error of the mean (n = 3); where error bars are not visible, variation is minimal and obscured by the marker. **Different (P ≤ 0.010) from the CN treatment

The presence of *C. rodentium* was not detected in the feces of mice inoculated with phosphate buffered saline (PBS). In contrast, mice in all treatment groups inoculated with *C. rodentium* exhibited the highest (P = 0.001) levels of shedding on days 3–10 p.i. (Fig. 1). Mice consuming the WB and RS diets shed the highest amount of *C. rodentium* on day 7 p.i., while mice ingesting the control (CN) diet shed the highest counts of *C. rodentium* on day 10 p.i. (Fig. 1). Mice consuming the WB diet shed *C. rodentium* at higher quantities than mice ingesting the CN (P = 0.004) and RS (P = 0.005) diets. Densities of *C. rodentium* in feces steadily decreased for all diet treatments after day 10 p.i. At the later stages of infection (>14 days p.i.), a trend (P = 0.168) of lower *C. rodentium* densities in the feces was observed in mice consuming the WB diet in comparison to the RS and CN diet treatment mice.

### Histopathologic changes and colon length in mice with enteritis

**Fig. 2:**
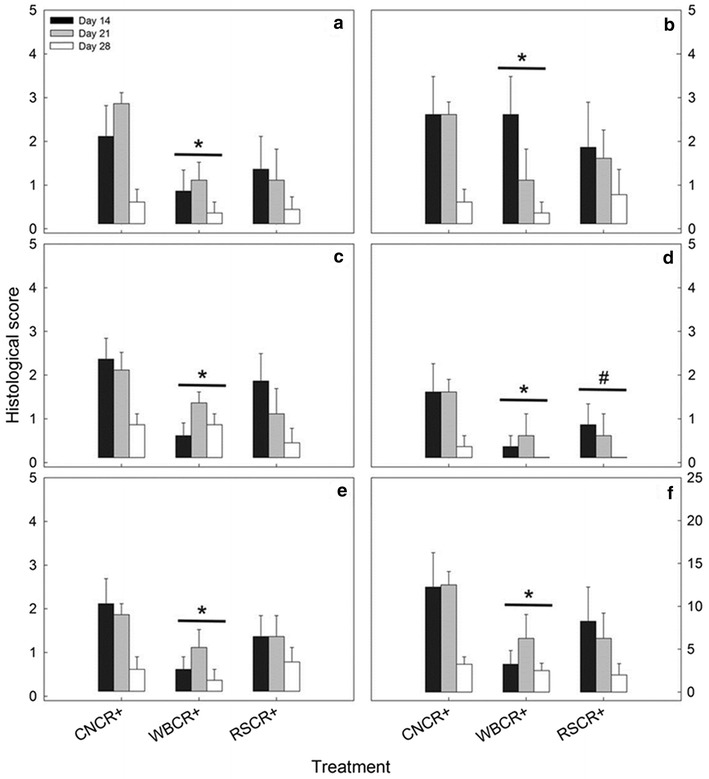
Histological scores measured in the proximal colons of mice inoculated with *C. rodentium* (CR+) consuming a control diet (CN), or a diet enriched with wheat bran (WB) or resistant starch (RS). **a** Epithelial cell hyperplasia. **b** Crypt height. **c** Epithelial cell injury. **d** Goblet cell depletion. **e** Mitotic cell activity. **f** Total average histological scores. *Vertical lines* associated with *histogram bars* represent the standard error of the mean (n = 4). ^#^Different (P ≤ 0.100) from the CN treatment. *Different (P ≤ 0.050) from the CN treatment. Statistical analyses are representative of scores averaged over all time points

**Fig. 3:**
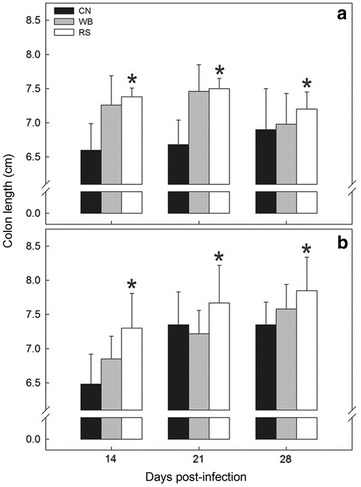
Average colon length (cm) in mice gavaged with PBS and inoculated with *C. rodentium* consuming a control diet (CN), or a diet enriched with wheat bran (WB) or resistant starch (RS) over a 28 day period. **a** Mice gavaged with PBS. **b** Mice inoculated with *C. rodentium*. *Vertical lines* associated with *histogram bars* represent the standard error of the mean (n = 4). *Different (P ≤ 0.050) from the CN treatment

Mice without enteritis exhibited total average histopathological scores of ≤1.75 ± 0.48 in the proximal colon, and ≤1.25 ± 0.63 in the distal colon; furthermore, these mice did not show clinical manifestations of intestinal disease or signs of infection (Additional file 1: Figure S1, S2). Mice inoculated with *C. rodentium* exhibited substantially higher histopathologic scores (P ≤ 0.001) relative to mice inoculated with PBS for all of the categories examined. Histopathologic evidence of acute inflammation incited by *C. rodentium* was observed in both the proximal (Fig. 2) and distal (Additional file 1: Figure S3) colon. The degree of cellular injury was highest on day 14 p.i. (i.e. peak infection) and day 21 p.i. (i.e. late infection), and decreased by day 28 p.i. (i.e. clearance) (Fig. 2a–f). Histopathologic scores of cellular injury in the distal colon were similar (P ≥ 0.322) among the diet treatments for mice with enteritis (Additional file 1: Figure S3A–G). However, in the proximal colon, mice fed the WB diet (P = 0.029) showed altered histological scores overall as compared to those fed the CN diet (Fig. 2a–f). Specifically, mice fed the WB diet displayed lower measures of epithelial cell hyperplasia (P = 0.035), goblet cell depletion (P = 0.018), epithelial cell injury (P = 0.027), mitotic activity (P = 0.032), crypt height (P = 0.024), and a lower total score of inflammation (P = 0.029) than the CN diet treatment mice. Mice fed the RS diet displayed a trend of reduced (P = 0.093) goblet cell depletion in comparison to mice consuming the CN diet. Averaged over time, mice consuming the RS diet also had longer (P = 0.015) colons in comparison to the CN and WB diet mice (Fig. 3). Overall, mice consuming diets high in DF displayed lower scores of inflammation in the proximal colon, in comparison to the distal colon.

### Food consumption and weight gain in mice

**Fig. 4:**
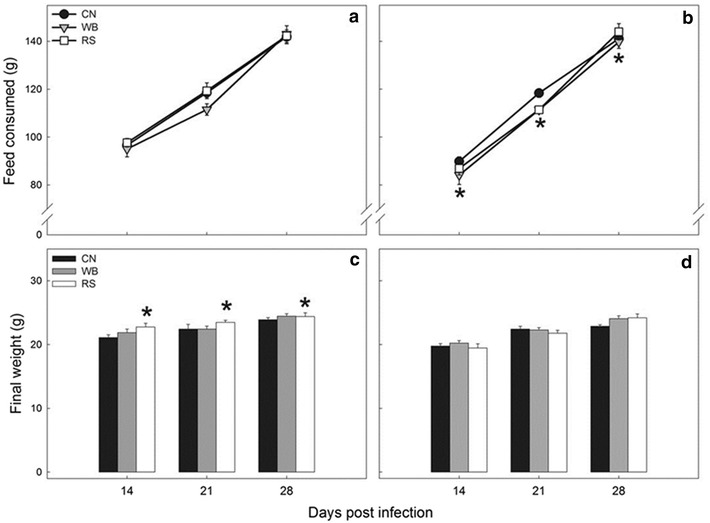
Feed consumption and final weight values in mice gavaged with PBS and inoculated with *C. rodentium* consuming a control diet (CN), or a diet enriched with wheat bran (WB) or resistant starch (RS). **a** Feed consumption in mice without enteritis. **b** Feed consumption in mice with enteritis. **c** Body weights of mice without enteritis. **d** Body weights of mice with enteritis. *Vertical lines* associated with *histogram bars* represent the standard error of the mean (n = 4) where error bars are not visible, variation is minimal and obscured by the marker. *Different (P ≤ 0.050) from the CN treatment

Diet treatment had no effect (P = 0.285) on the amount of food consumed in mice without enteritis (Fig. 4a). However, mice with enteritis administered the WB diet consumed less (P = 0.030) food than those fed the CN diet (Fig. 4b). In all mice, the average final weights of mice steadily increased (P < 0.001) over the 28-day experimental period (Fig. 4c, d). In mice without enteritis (Fig. 4c), individuals consuming the RS diet gained weight more rapidly (P = 0.017) than those consuming the CN diet. Mice with enteritis gained less (P < 0.001) weight overall in comparison to mice without enteritis.

### Short-chain fatty acid concentrations in the cecum and distal colon

**Fig. 5:**
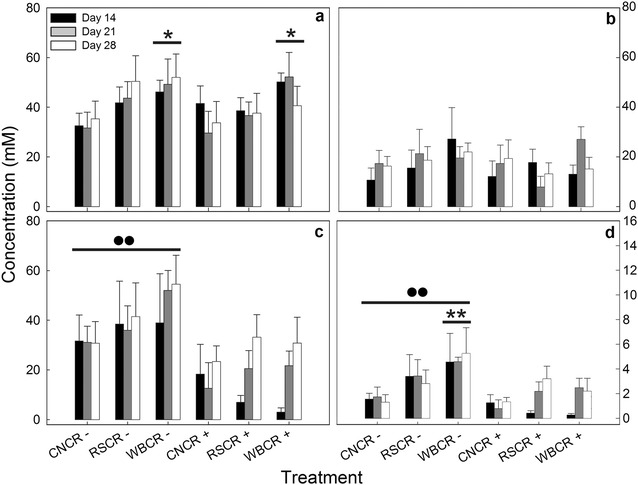
Total short-chain fatty acid (SCFA) and butyrate concentrations in the cecum, proximal colon and distal colons of mice gavaged with PBS (CR−) and inoculated with *C. rodentium* (CR+) and consuming the control diet, or a diet enriched with wheat bran (WB) or resistant starch (RS). **a** Total SCFA concentrations in the cecum. **b** Total SCFA concentrations in the proximal colon. **c** Total SCFA concentrations in the distal colon. **d** Butyrate concentrations in the distal colon. *Vertical lines* associated with *histogram bars* represent the standard error of the mean (n = 4). *Different (P ≤ 0.050) from the corresponding CN treatment. ^●●^Difference (P ≤ 0.010) relative to CR+ mice

Short-chain fatty acid concentrations were measured in the cecum, proximal colon, and distal colon, and quantities of total SCFAs differed between these locations (Fig. 5a–c). Generally, the highest quantities of total SCFAs were observed in the cecum and distal colon. Concentrations of total SCFAs (P < 0.001), and butyrate specifically (P = 0.003) were higher in mice without enteritis than in mice with enteritis in the distal colon (Fig. 5c, d). In the cecum, enteritis had no effect on total SCFA concentrations (P = 0.497) (Fig. 5a). However, total SCFA (P = 0.008) (Fig. 5a), acetate (P = 0.013) (Additional file 1: Table S1), and butyrate (P < 0.001) (Additional file 1: Table S1) were all affected by the diet treatment. Butyrate concentrations in the cecum were higher for the WB diet than the CN (P < 0.001) and RS (P = 0.009) diet treatments (Additional file 1: Table S1). In the proximal colon, mice fed the WB diet exhibited higher (P = 0.051) overall concentrations of SCFAs as compared to those fed the RS and CN diets (Fig. 5b) (Additional file 1: Table S1). In the distal colon, concentrations of total SCFAs (P < 0.001), acetate (P < 0.001), propionate (P = 0.002), and butyrate (P = 0.003) were substantially reduced in mice with enteritis at the peak, late, and clearance stages of infection (Fig. 5c) (Additional file 1: Table S1). Furthermore, mice consuming the WB (P = 0.005) and RS (P = 0.053) diets showed higher butyrate concentrations as compared to those consuming the CN diet (Fig. 5a–c). In mice without enteritis, butyrate concentrations in mice consuming the WB diet were higher (P = 0.001) relative to the CN diet treatment in the distal colon (Fig. 5d) (Additional file 1: Table S1).

### Mucus accumulation

**Fig. 6:**
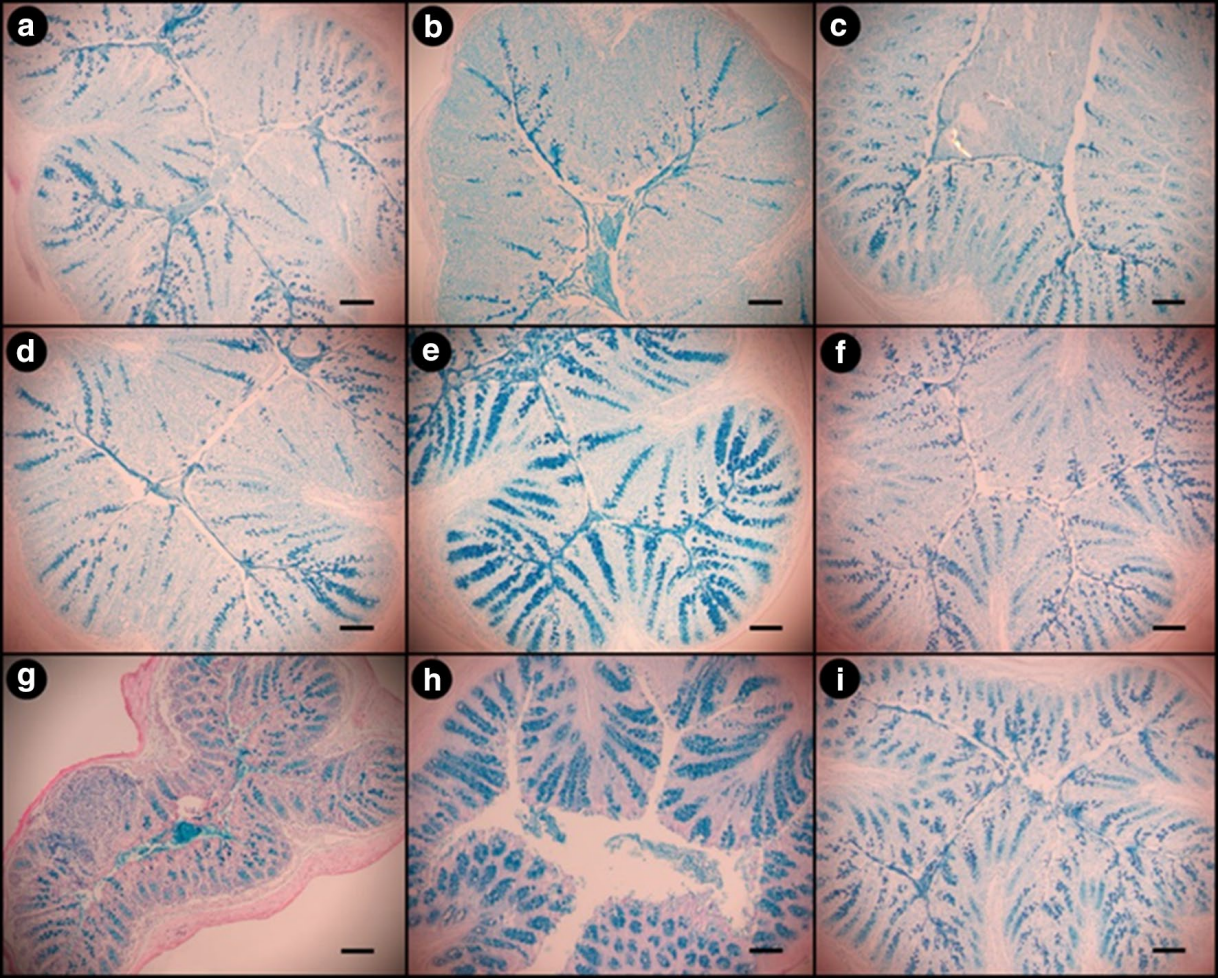
Mucus in the distal colons of mice inoculated with *C. rodentium* consuming a control diet (CN), or a diet enriched with wheat bran (WB) or resistant starch (RS). **a** CN treatment on day 14 post-inoculation (p.i.), **b** WB treatment on day 14 p.i., **c** RS treatment on day 14 p.i., **d** CN treatment on day 21 p.i., **e** WB treatment on day 21 p.i., **f** RS treatment on day 21 p.i., **g** CN treatment on day 28 p.i., **h** WB treatment on day 28 p.i., **i** RS treatment on day 28 p.i. *Alcian Blue stain* identifies mucus staining within the colonic sections. Bar, 100 µm

**Fig. 7:**
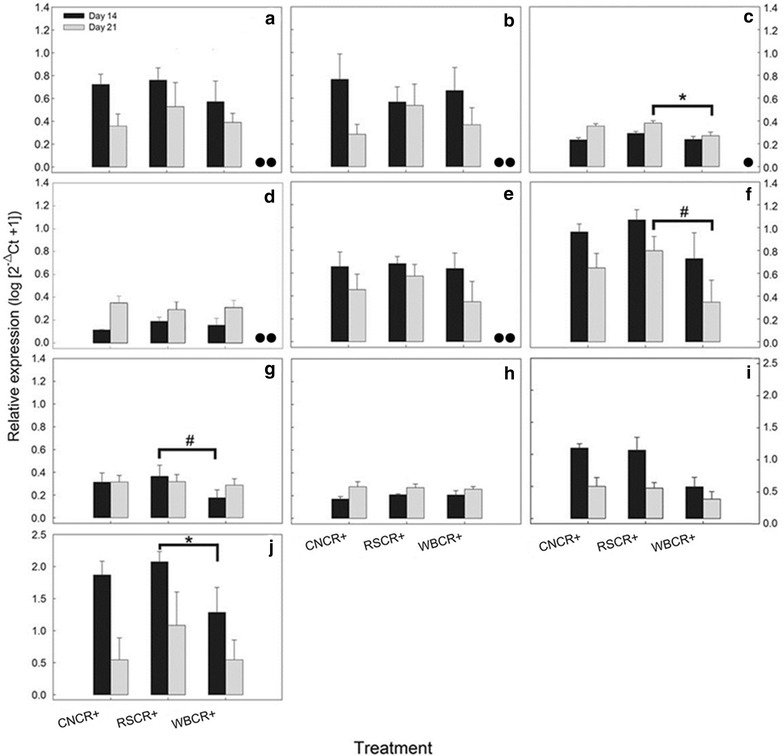
Expression of genes involved in the Th17, Th1, and Treg immune response as well as bacterial recognition and cellular repair genes from the distal colon of mice inoculated with *C. rodentium* (CR+) consuming a control diet (CN), or a diet enriched with wheat bran (WB) or resistant starch (RS). **a** *Il*-*17A*, **b** *Il*-*1β*, **c** *Tlr4*, **d** *Muc2*, **e** *Tnfα*, **f** *Ifnγ*, **g** *Il*-*10*, **h** *Tgfβ*, **i** *RegIIIγ*, **j** *Relmβ*. *Veritcal lines* associated with *histogram bars* represent the standard error of the mean (n = 3). *Pairwise differences (P ≤ 0.050) between treatments. ^#^Pairwise differences (P ≤ 0.100) between treatments. ^●●^Difference (P ≤ 0.010) in overall expression compared to non-inflamed gene expression (Additional file 1: Figure S5). ^●^Difference (P ≤ 0.100) relative to non-inflamed gene expression

The accumulation of mucus in the distal colon of mice without enteritis did not differ between the diets (Additional file 1: Figure S4). Mucus accumulation in goblet cells and to a lesser extent within the intestinal lumen of mice with enteritis was marginally increased for mice consuming the WB diet (Fig. 6b, e, h) as compared to the CN diet (Fig. 6a, d, g). Increased mucus accumulation in the goblet cells was also observed in mice consuming the RS diet (Fig. 6c, f, i) relative to the CN diet (Fig. 6a, d, g) at the clearance stage of infection. Mice with enteritis exhibited reduced expression of *Muc2* as compared to mice without enteritis (P < 0.001) (Fig. 7d) (Additional file 1: Figure S4). At peak infection, it was observed that RS diet treatment mice exhibited the highest (P = 0.043) expression of *Muc2* in the distal colon (Fig. 7d) as compared to the CN diet; however by late infection, there was no difference (P = 0.387) between the two treatments.

### Expression of immune regulation and bacterial recognition genes

In mice without enteritis, there was no effect (P ≥ 0.100) of diet on the expression of Th1 (*Il*-*1β*, *Ifnγ*, *Tnfα*) or Th17 (*Il*-*17A*, *Il*-*22*, *Il*-*23A*) associated cytokines, Treg (*Il*-*10*, *Tgfβ*), mucus associated (*Muc2*, *Tff3*, *Relmβ*), or bacterial recognition (*Tlr2*, *Tlr4*, *Myd88*, *RegIIIγ*) genes (Additional file 1: Figure S5). In mice with enteritis, Th17 (*Il*-*17A*) and Th1 (*Il*-*1β*, *Tnfα*) associated cytokine genes did not show changes in expression in response to DF-enriched diets, but were highly expressed as compared to mice without enteritis (Fig. 7a, b, e). At peak infection, mice consuming the RS and WB diets exhibited the highest expression (P = 0.040) of *Tlr4* relative to CN diet treatment mice (Fig. 7c). For the RS diet treatment, a trend of increased (P = 0.083) *Tlr4* expression was observed in mice with enteritis relative to those consuming the WB diet. Mice consuming the RS diet also exhibited a trend towards elevated (P = 0.083) expression of *Ifnγ* during late infection, and increased (P = 0.043) expression of the defence cytokine, *Relmβ* at peak infection as compared to mice consuming the WB diet (Fig. 7f, j). Although not statistically significant (P > 0.050), mice consuming the RS diet tended to exhibit elevated expression (P ≤ 0.083) of Treg cytokines (i.e. *Il*-*10*, *Tgfβ*), and this was evident during peak infection as compared to mice consuming the WB and CN diets, respectively (Fig. 7g, h). The expression of *RegIIIγ* was highest (P = 0.016) at peak infection in all mice, and those consuming the WB diet exhibited the lowest expression of this gene (P = 0.021) relative to mice consuming the CN diet (Fig. 7i). Generally, mice consuming the WB diets displayed lower expression of bacterial recognition genes (*Tlr4*, *RegIIIγ*), the defense cytokine gene *Relmβ*, the Th1 regulatory cytokine gene *Ifnγ*, and the regulatory cytokine gene *Il*-*10* during peak and late infection.

### Bacterial community structures in the large intestine

**Fig. 8:**
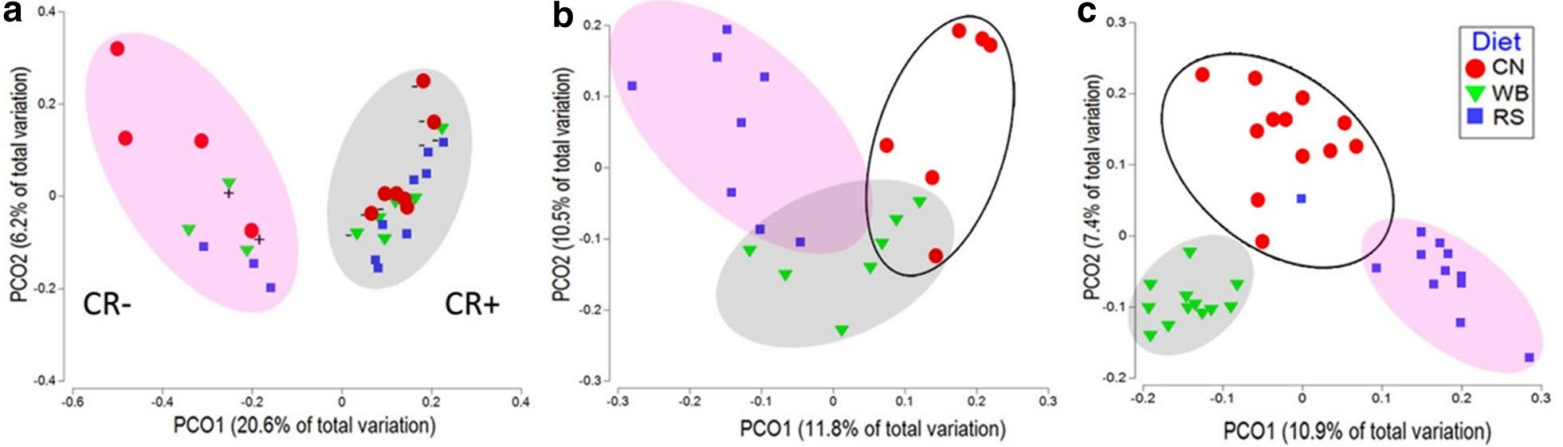
Unweighted Uni-Frac comparisons of the bacterial communities in the intestines of mice gavaged with PBS (CR−) and inoculated with *C. rodentium* (CR+) consuming a control (CN) diet, or a diet enriched with wheat bran (WB) or resistant starch (RS). **a** Distal colon. Ellipsoids identify clusters of communities by infection treatment. **b** Proximal colon. Ellipsoids identify clusters of communities by diet treatment. **c** Cecum. Ellipsoids identify clusters of communities by diet treatment

The bacterial communities associated with mucosa in the proximal and distal colon, and within ingesta in the cecum were distinct from one another. Both enteritis and diet affected community structure (Fig. 8). The DF treatments were associated with unique community structures in the proximal colon (P = 0.001), and within cecal ingesta (P = 0.001) (Fig. 8b, c). Although less conspicuous than in the proximal colon, diet also affected (P = 0.024) the structure of the mucosa-associated community in the distal colon (Fig. 8a). Within the distal colon, enteritis had a profound impact (P = 0.006) on bacterial communities (Fig. 8a).

**Fig. 9:**
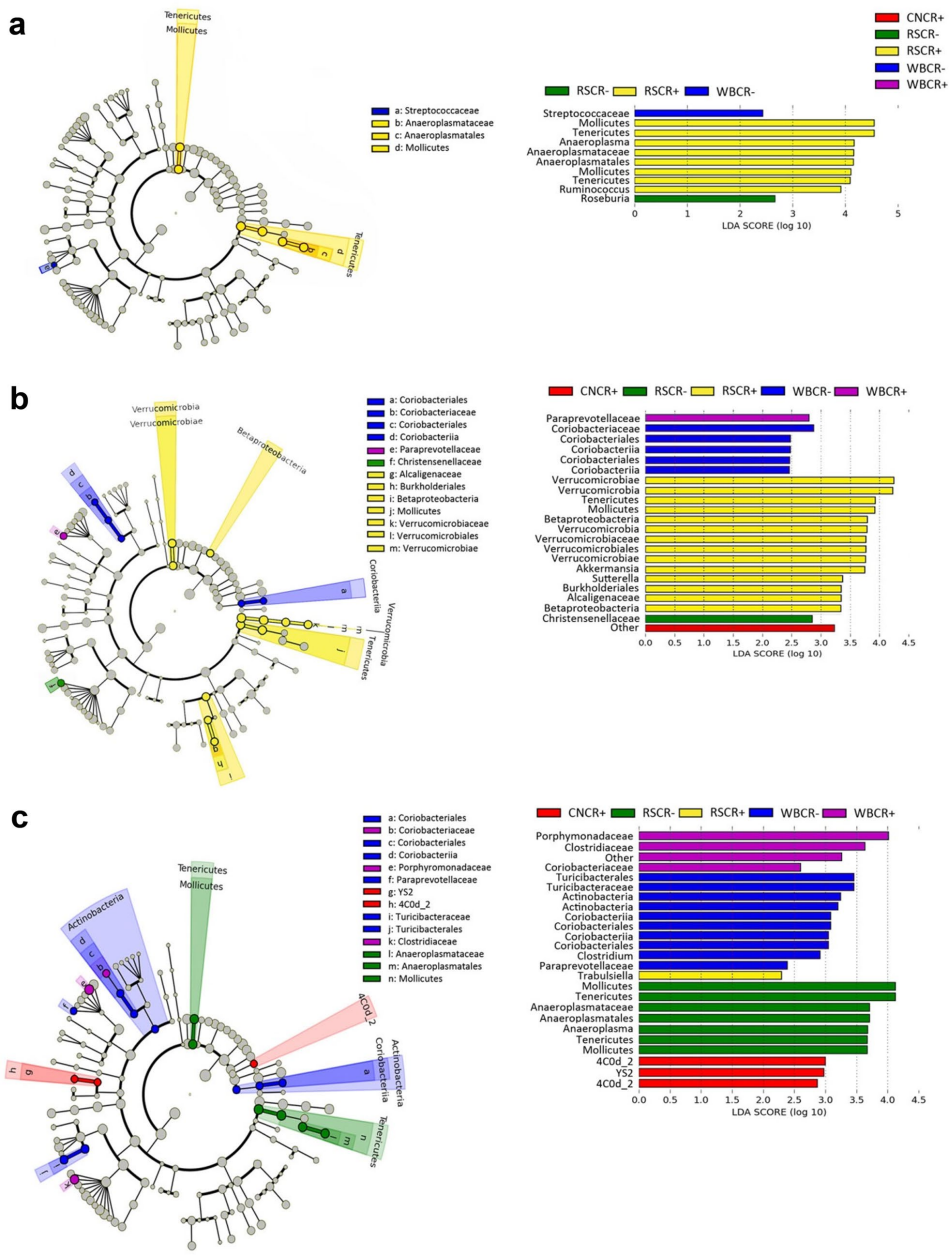
Cladograms identifying bacterial taxa that are differentially abundant and biologically consistent in mice gavaged with PBS (CR−) and inoculated with *C. rodentium* (CR+), comparing the differences between mice consuming a control (CN) diet, or a diet enriched with wheat bran (WB) or resistant starch (RS). **a** Communities associated with mucosa in the distal colon. **b** Communities associated with mucosa in the proximal colon. **c** Communities within cecal ingesta. Highlighted taxa represent significantly (P ≤ 0.050) impactful communities within each treatment

**Fig. 10:**
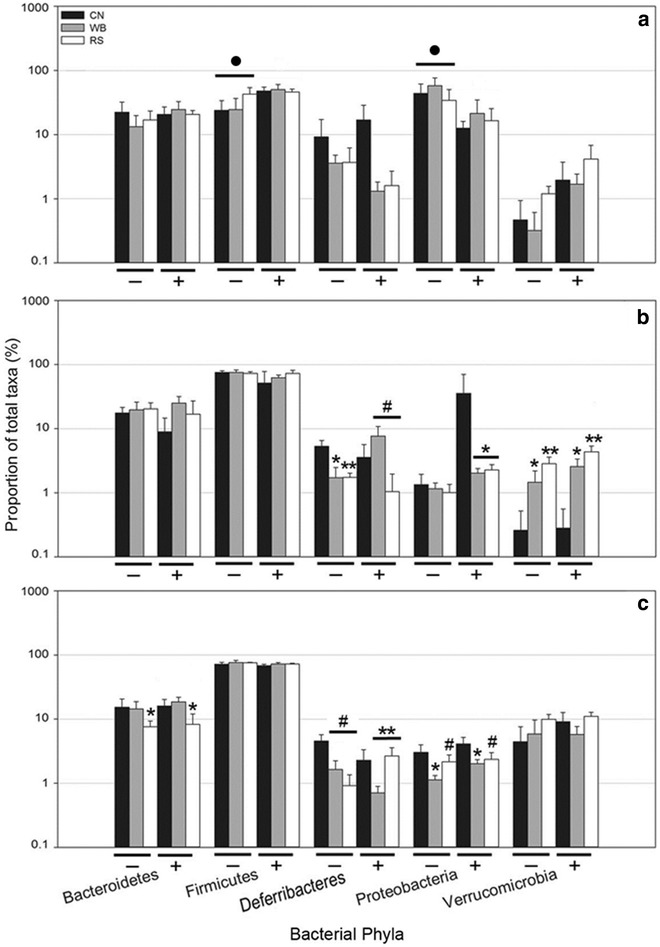
Proportion of bacterial taxa in the common phyla in mice gavaged with PBS (−) and inoculated with *C. rodentium* (+) and consuming a control (CN) diet, or a diet enriched with wheat bran (WB) or resistant starch (RS). **a** Bacterial abundance associated with the distal colon. **b** Bacterial abundance associated with mucosa in the proximal colon. **c** Bacterial abundance in the cecal ingesta. *Vertical lines* associated with *histogram bars* represent the standard error of the mean (n = 6). ^●^Different (P ≤ 0.050) relative to the CR+ treatment. ^#^Different (P ≤ 0.100) relative to the CN treatment. *Different (P ≤ 0.050) relative to the CN treatment. **Different (P ≤ 0.010) relative to the CN treatment

In contrast, *C. rodentium* infection did not alter (P > 0.658) community structures in the proximal colon or cecum. The consumption of the RS diet was associated with an increase (P = 0.005) in the abundance *Roseburia* spp. in the distal colon of mice without enteritis, and an increase (P = 0.034) in the abundance of *Ruminococcus* spp. in the distal colon of mice with enteritis (Fig. 9a) in comparison to mice consuming the CN diet. The abundance of *Proteobacteria* in the distal colon was lower (P = 0.016) in mice with enteritis as compared to mice without enteritis (Fig. 10a). In mice consuming the RS diet, an increase (P = 0.001) in the abundance of *Verrucomicrobia* and the mucus-associated species, *Akkermansia muciniphila* was observed in the proximal colon (Fig. 9b). In addition, the abundance of *A. muciniphila* was increased (P = 0.045) in the proximal colon of mice that consumed the WB diet, and most notably in mice with enteritis (Fig. 10b). Furthermore, an increase (P < 0.001) in the abundance of bacterial species within the family, *Christensenellaceae* was observed in the proximal colon of mice without enteritis that consumed the RS relative to the CN diet (Fig. 9b). In the cecal ingesta of mice that ingested the RS diet, the abundance of species in the *Bacteroidetes* phylum was reduced (P = 0.055) relative to the CN diet (Fig. 10c). However, in mice without enteritis that consumed the RS diet, a conspicuous increase (P ≤ 0.050) in the abundance of species in the *Actinobacteria* phylum was observed (Fig. 9c). The consumption of the WB diet was associated with a change (P ≤ 0.050) in the abundance of bacteria within the *Actinobacteria* (i.e. *Coriobacteriaceae*) and *Bacteroidetes* (i.e. *Porphyromonadaceae* and *Paraprevotellaceae*) phyla in comparison to mice consuming the RS and CN diets (Fig. 9c). It is noteworthy that all species within the *Verrucomicrobia* phylum were identified as *A. muciniphila*, while all species identified within the *Deferribacteres* phylum were identified as *Mucispirillum schaedleri*. As such, a general trend of reduced *Deferribacteres* and *M. schaedleri* abundance was observed in the cecal ingesta of mice with enteritis that consumed both the WB and RS diets; however, the mucus degrading *A. muciniphila* showed a trend of increased abundance in mice without enteritis that consumed either the WB or RS diets (Fig. 10c). Finally, the abundance of butyrogenic species including *Dorea*, *Ruminococcus*, and *Roseburia* spp. was increased in the ceca and colons of mice with and without enteritis that consumed diets enriched with WB or RS (Additional file 1: Figure S6).

## Discussion

Understanding the mechanisms by which functional foods such as DFs influence the microbiota-host interaction is important to identify food components that can potentially improve host health. This is a challenging objective given the requirement to use animal models, and the extreme complexity of the diet-microbiota-host interaction. Our investigation examined the effects of DFs on the mitigation of intestinal injury in mice with enteritis. We used mice fed diets enriched with RS and WB that were subjected to pathogen challenge with *C. rodentium* to induce inflammation. Our results demonstrated that DFs alter bacterial communities within the large intestine, increase total SCFA concentrations in the cecum and colon, reduce inflammation, and improve weight gain.

### Modulation of weight gain and appetite

Healthy mice consume more food and proportionally gain more weight in comparison to mice experiencing intestinal inflammation induced by chemical and intestinal pathogens [39–41]. WB consumption triggers stimuli that promote satiation within the host [42], and we observed a consistent decrease in food intake by mice with and without enteritis consuming the WB-enriched diet. RS-enriched diets have been reported to increase levels of glucagon-like pepetide-1 and peptide YY, which are secreted hormones that reduce appetite and food intake in human and non-human animals [43]. These hormones act in response to ingested nutrients, and both RS and WB diets are triggers to their secretion [43, 44]. Although we observed that food consumption was reduced in mice fed DF-enriched diets during infection, the final weights of these mice did not differ among the diets. Many studies have supported the conclusion that consumption of DF reduces food consumption and obesity in human beings [45–47]. In our study, mice with enteritis did not show a significant reduction in weight gain. A plausible explanation is that under conditions of enteric inflammation, mice generally gain less weight and have less efficient nutrient absorption due to cellular injury [48]. We observed that mice without acute enteritis that consumed a RS-enriched diet had higher final weights, and a longer colon; the increased body weight and length of the colon is consistent with an increase in the combined mass of the intestinal cells [49]. This may not only translate to enhanced anabolism, but improved mucosal repair, barrier function and enterocyte hyperplasia, contributing to an increase in colon length are well-recognized manifestations of mitigated intestinal inflammation [40]. Although DF intake can reduce feed intake, generally, the total amount of calories required for growth is not affected, and individuals can still maintain a healthy weight [50]. Furthermore, highly fermentable fibers may accelerate fecal passage time; although decreased transit time can impart negative effects on the physiology of the host, it may also contribute to increased shedding and removal of bacterial pathogens [39, 51, 52]. Taken together, evidence indicates that the consumption of diets enriched with WB and RS reduces the amount of food necessary to maintain a healthy body weight in mice.

### Alteration of infection severity by wheat bran and cellular inflammation by resistant starch

Mice inoculated with *C. rodentium* exhibit transmural crypt hyperplasic changes within the distal colon, and although tissue injury is profound, the infection is resolved 3–4 weeks p.i. [25]. We observed that mice consuming the WB-enriched diet shed more colony-forming units (CFU)/g in feces during early infection, but by peak and late infection, mice shed less CFU/g as compared to those consuming the RS-enriched and CN diets. Shedding of *C. rodentium* can be affected by the carbohydrate composition of diets. In one study, diets enriched with both digestible carbohydrates and non-digestible fiber demonstrated reduced clearance of *C. rodentium,* an observation linked to altered adaptive immunity and intestinal bacterial community structures [40]. *C. rodentium* infection initially elicits a predominantly Th17 immune response that switches to a Th1 response as infection progresses [27]. In mice consuming the WB-enriched diet, the expression of the Th17 cytokine, *Il*-*17A* was lower than mice consuming the RS-enriched and CN diets during peak infection, and by late infection, these mice exhibited the lowest expression of Th1 (*Il*-*1β*, *Ifnγ*, *Tnfα*) cytokines. Furthermore, the overall amount of *C. rodentium* shed in feces was the lowest in mice consuming the WB-enriched diet at late infection. This suggests that mice ingesting the WB-enriched diet were less affected by infection relative to mice consuming the RS-enriched and CN diets, and that the administration of WB-enriched diets can reduce intestinal inflammation and the host immune response by undetermined compound(s) that are independent of SCFA production [53]. Further research is needed to identify the products of WB fermentation that improve intestinal function and health. Measures of tissue injury and cellular inflammation in the colon indicated that infection in the proximal colon was less severe as compared to the distal colon in the current study. As *C. rodentium* mainly causes damage to the distal colon [25], we conclude that the ingestion of WB is ameliorative to inflammation in tissues that are less severely inflamed (i.e. in the proximal colon). Furthermore, it has been shown that DF administration does not influence the growth of invasive pathogens such as enteropathogenic *Escherichia coli* and *C. rodentium* through elevated Treg (*Il*-*10*, *Tgfβ*) cytokine expression [38]. We observed that genes indicative of bacterial infection and inducers of inflammation, namely *Tlr4* and *RegIIIγ* were expressed at lower amounts in mice consuming the WB-enriched diet compared to RS-enriched diet. TLR4 is involved in increasing the pro-inflammatory immune response through the recognition of bacterial lipopolysaccharide [54], and RELMβ is involved in promoting protective epithelial cell proliferation during *C. rodentium* infection in mice [55]. We also observed elevated expression of *Relmβ* in the distal colons of mice consuming the RS-enriched diet in concert with high scores of epithelial cell hyperplasia, suggesting that the heightened histopathologic scores observed in the distal colon were a result of protective cell growth rather than destructive cell growth [55]. Furthermore, in mice gavaged with PBS alone, those consuming the RS-enriched diet exhibited elevated levels of *Relmβ* as well as an increase epithelial cell hyperplasia in the distal colon compared to mice consuming the WB-enriched diet. Overall, the consumption of DFs, namely WB was beneficial in reducing the severity of *C. rodentium* infection in the proximal colon, and this was confirmed by the lowered expression of genes that are hallmarks of inflammation. In the distal colon where infection is more severe, DF was less effective at reducing pathologic changes; however, the RS-enriched diet stimulated innate factors (i.e. *Relmβ*) to enhance the host immune response and increase cell proliferation to reduce tissue damage. In the current study, we limited our examinations to intestinal tissues and the examination of markers in serum (e.g. cytokines) may yield extra-intestinal information on the impacts of DF digestion on enteric inflammation.

### Intestinal short-chain fatty acids in relation to butyrogenic and mucus-associated bacteria

Intestinal SCFAs are important in the maintenance of colonic function and intestinal homeostasis. Accurate measurements of colonic SCFAs can be challenging as they are rapidly metabolized by epithelial cells in the large intestine [56]. Fermentation rates are highest in the cecum and proximal colon [57], yet our data shows that the proximal colon had the lowest quantities of SCFAs of the three sites examined. This suggests that SCFAs may be absorbed at a faster rate in the proximal colon than in the distal colon and cecum [10, 58]. In the proximal colon, we observed no differences in SCFA concentrations between mice with and without acute enteritis. In contrast, SCFA concentrations were higher in the distal colon of mice without enteritis. Amongst these mice, SCFA concentrations in the distal colon were elevated in mice that consumed DF-enriched diets, and those ingesting the WB-enriched diet specifically showed the highest concentrations of SCFAs. Collectively, our results suggest that DF consumption is most effective at increasing total SCFA and butyrate concentrations and contributing to host health in the non-inflamed or moderately inflamed colon [10].

An increase in enteric concentrations of SCFAs is purported to confer a variety of health benefits, and individually each SCFA may elicit positive effects on the intestine [4, 59, 60]. Butyrate in particular is an important SCFA in the colon. Butyrate is the primary energy source for colonocytes, it improves mucosal barrier function, by enhancing the expression of proteins involved in tight junctions, and induces the development of colonic Treg T cells [61–63]. Butyrate is also associated with increasing intestinal mucus production [64–66], and although studies are conflicting, many suggest a positive correlation between butyrate presence and mucin secretion. Although we did not observe changes in *Muc2* expression in response to diet treatment, we did observe that mice consuming the WB-enriched diet exhibited more mucus within goblet cells. Of the diet treatments that we examined, the WB-enriched diet continuously produced the highest concentrations of total SCFAs, including butyrate. Previous studies have revealed that β-glucan can contribute to enhanced mucus secretion in the intestine [67]. Furthermore, others have shown that diets enriched for long-chain arabinoxylans and inulin increase the concentration of SCFAs in the colons of mice, and concomitantly increase host-derived mucins and butyrogenic bacteria [68]. Although RS fermentation is known to increase intestinal butyrate concentrations [14], arabinoxylan fractions found in WB can also increase butyrogenic bacteria, and contribute to higher butyrate concentrations within the colon than would be produced via fermentation of RS alone [22]. In this regard, the highest concentrations of butyrate were observed in feces from human subjects that consumed wheat arabinoxylan over a 3-week period [69]. Although we did not observe conspicuous effects of RS and WB diet enrichment on host metrics in mice without enteritis (e.g. immune responses), it is possible that the by-products of DF fermentation could play an important role in maintaining physiological homeostasis in the quiescent intestine. This suggests that improved intestinal health and growth can occur through the maintenance of the physiologic, metabolic, and immunological balance within the intestinal mucosa, and would reduce unwanted energy loss from enhanced catabolic demands due to ‘physiologic intestinal inflammation’ [70]. Our results of immune modulation in mice afflicted by acute enteritis suggest that DF consumption may also contribute to host health in the non-inflamed or moderately inflamed colon [10].

An increase in the abundance of mucus-associated bacteria in the colon of mice was consistently observed in the current study, especially in mice with enteritis. The abundance of bacterial species known to be associated with mucus can be indicative of high mucus content within the intestine. *Mucispirillum shaedleri* is recognized as a mucus-dwelling bacterium [71, 72]. However, the degree and mechanisms by which *M. shaedleri* degrades mucus, and the importance of mucus as an ecological niche for this bacterium remains to be elucidated. Alternatively, *A. muciniphila* has been widely reported as a mucin degrader, and it utilizes gastric mucin as its main energy source in healthy individuals [73, 74]. We observed that the abundance of the mucus-dwelling bacterium, *M. schaedleri* in the proximal and distal colon (mucosa-associated) and cecum (ingesta) was decreased in healthy mice consuming DFs. Conversely, *A. muciniphila* abundance was increased in the colon of mice administered DFs, and this was observed in all mice regardless of their inflammation status. This suggests that during infection, the increase in *A. muciniphila* abundance decreases the mucus available for colonization by *M. schaedleri* (i.e. due to increased mucus degradation by *A. muciniphila*). We did not detect elevation of *Muc2* expression in the distal colon as a function of diet treatment. It is plausible that other intestinal mucins such as MUC4 or MUC5B may have increased in the intestines of mice consuming the DF-enriched diets other than MUC2 [67]. The composition, architecture, and microbiology of mucus is complex [75, 76], and the impact of DFs on the biochemical and microbiological characteristics of enteric mucus warrants examination.

### Microbial community structures in mice with and without enteritis

The consumption of DFs altered the structure of bacterial communities in the distal colon, proximal colon, and cecum. We observed that enteritis affected communities in the distal colon to a greater degree than DF enrichment of diets, and mice consuming the RS-enriched diet exhibited an increase in the abundance of *Firmicutes*, which has been previously reported in mice infected with *C. rodentium* [77]. Our experiment also demonstrated that mice challenged with *C. rodentium* had reduced numbers of *Proteobacteria* within the proximal colon, an interesting and unexpected observation. Research has shown that *C. rodentium* infection will increase species belonging to the *Proteobacteria* phylum in mice fed standard chow diets [77]. The discrepancy between studies suggests that components within the diet alter the intestinal microbiota composition. Indeed, investigators showed that diets fortified with lavender extract decreased the abundance of *Proteobacteria* in mice infected with *C. rodentium* [77], an observation linked to the exclusion of *Proteobacteria* by bacteria within the *Firmicutes* phylum [78]. A number of butyrogenic bacteria (e.g. members of the *Lachnospiraceae* and *Ruminococcaceae* families within the *Clostridiales* order) reside in the colon [79], and we observed an increase in the abundance of butyrate-producing bacteria including *Roseburia* spp. within the *Clostridia* XIVa group in mice that ingested DF-enriched diets. Moreover, diets containing DFs have been shown to increase butyrate levels within the intestine; a process associated with changes in the composition of the microbiota [80]. Intestinal pH may stimulate the growth of butyrogenic bacteria [81]. The lower pHs (≈pH 5.5) that occur in the cecum and proximal colon due to SCFA production has been shown to enhance the growth of bacteria in *Clostridia* cluster IV and *Clostridia* cluster XIVa [82]. Thus, the increases in SCFA concentrations that we observed may be due to the increased abundance of butyrogenic bacteria in the colon and cecum [57]. We also observed that the proximal colon had a greater diversity of mucosa-associated bacteria in comparison to the distal colon. In the proximal colon of mice without enteritis, consumption of the RS-enriched diet was associated with an increased abundance of *Christensenella* spp. In contrast, the abundance of *A. muciniphila* was increased in mice with enteritis that consumed the RS-enriched diet. Both these species have been shown to improve barrier function [83], as well as weight maintenance and health in human beings [84, 85]. In the cecum of mice without acute enteritis that consumed the WB-enriched diet, we observed that the abundance of *Paraprevotella* and *Clostridium* spp. increased, which is similar to observations reported in human beings [86]. Thus, our data indicates that mice fed diets enriched with DFs exhibit enhanced growth of bacteria that thrive in low pH environments and produce butyrate as a metabolic by-product of DF fermentation. Although we did not observe an association between SCFA concentration and the intensity of inflammation in the distal colon, the consumption of DF did alter the structure of the bacterial community, and in particular contributed to the increase in mucus-associated species in areas of less intense inflammation, such as the proximal colon. Furthermore, an increase in the abundance of mucus-associated species may be associated with the regulation of host growth and weight gain, and may contribute to enhancing barrier function and maintenance to improve host health [83, 85, 87]. Intestinal fermentation is exceptionally complex biochemically and ecologically, and varies spatially and temporally within the large intestine. Branched-chain fatty acid fermentation in the distal colon increases ammonia and nitrogen substrates that can also affect bacterial community composition [31], yet the impacts of nitrogen metabolism resulting from fermentation on intestinal health has not been extensively studied.

## Conclusions

The consumption of DF has been promoted to improve intestinal health. In this study, we explored the influence of the DFs WB and RS on SCFA production, modulation of the intestinal microbiota, and intestinal health using a *C. rodentium* model of colitis in mice [77]. Although some differences in responses between RS and WB were observed, in general, DF was associated with elevated SCFA levels including butyrate within the large intestine, and reduced tissue inflammation and enhanced epithelial cell repair in inflamed intestines. DFs were also associated with an altered composition of the microbiota, favoring an increase in butyrogenic bacteria. Collectively, study findings indicate that DFs have the potential to improve intestinal health via a number of mechanisms.

## Methods

### Experimental design

The experiment was designed as a completely randomized design with three levels of diet (CN, RS, and WB), two levels of immunological stress (± *C. rodentium*), and three levels of sample time (14, 21, and 28 days p.i.). Each replicate included 36 mice, and four replicates were conducted on separate occasions (144 animals in total). It is noteworthy that separate mice were used for the quantification of SCFAs (n = 18 mice/replicate).

### Ethics statement

The study was carried out in strict accordance with the recommendations specified in the Canadian Council on Animal Care Guidelines. The project was reviewed and approved by the Lethbridge Research and Development Centre (LRDC) Animal Care Committee (Animal Use Protocol Review 1405), and the LRDC Biosafety and Biosecurity Committee before commencement of the research.

### Mouse maintenance

Specific pathogen free C57BL/6J mice were obtained from Charles River Laboratories (Montreal, QC) at 3-weeks of age. For each of the four replicates, mice were group-housed with six mice per cage upon arrival, and were given 5 days to acclimate under a 10:14 h dark:light cycle. After adaptation, mice were individually housed and permitted to eat and drink ad libitum. Cages were lined with sterile bedding and housing units, and along with the food and water were replaced weekly. Mice were monitored daily to confirm their health status [88], and initial body weights were taken prior to oral inoculation with *C. rodentium* in PBS (0.01 M NaH_2_PO4, 0.04 M Na_2_HPO4, 0.07 M NaCl, Sigma-Aldrich; pH 7.4) or PBS alone. Body weights were measured at the time of euthanization to ascertain total weight gained during the experimental period. Feed was also weighed on the day of inoculation and at euthanization to determine the total weight of food consumed.

### Dietary fiber supplementation

Upon arrival at LRDC, mice were maintained on a conventional rodent chow diet. After the 5-day adaptation period, mice were switched to experimental diets for a period of 14 days prior to inoculation with *C. rodentium* or PBS alone. The basal diet consisted of a modified AIN-93G purified rodent diet with sterile vitamin free casein (DYET#103455GI). For the WB diet, the basal diet was enriched with 117 g/kg of Bob’s Red Mill WB (DYET #103456GI). For the RS diet, the basal diet was enriched with 125 g/kg of King Arthur Flour Hi-Maize^®^ (i.e. type 2 RS) (DYET#103457GI). The CN diet consisted of the basal diet alone. All diets (Bethlehem, PA, USA) were color coded with dye (i.e. to ensure accurate administration), pelleted, vacuum sealed and irradiated, and stored at −20 °C for short periods until used.

### *Citrobacter rodentium* inoculation

A green fluorescent protein (GFP)-labelled *C. rodentium* DBS100 (ATCC 51459) was used. The bacterium was grown aerobically at 37 °C for 24 h on Lysogeny Broth agar (LA) with 30 µg/mL chloramphenicol [65]. In a preliminary study, it was determined that neither RS or WB affected growth of *C. rodentium* (data not presented). Bacterial cells for inoculation were grown in sterile Lysogeny Broth (LB) containing 15 µg/mL chloramphenicol (Sigma-Aldrich, Oakville, ON) for 2 h at 37 °C at 100 rpm, until an OD_600_ > 0.1 was obtained. Cells were pelleted by centrifugation at 2256×*g* for 15 min, the supernatant was removed, and cells were re-suspended in 3.0 mL of PBS. Cell density and viability was confirmed using a tenfold dilution series, and 100 µL of each dilution was spread in duplicate onto LA, and cultures were incubated at 37 °C. Cell densities were adjusted to 2 × 10^9^ CFU/mL with PBS. Eighteen mice were gavage inoculated with *C. rodentium* cells (100 µL) using a 22G × 2.5 cm-long gavage needle with a 1.25 mm ball tip on two consecutive days. Similarly, 18 mice were gavage inoculated with PBS alone (100 µL). Animals were monitored for discomfort/pain for 4 h after inoculation using a quantitative pain assessment scoring system as specified by the LRDC Animal Care Committee.

### Isolation of *C. rodentium* from collected feces

Fecal samples from mice were collected 3, 7, 10, 14, 21, and 28 days p.i. Briefly, fecal samples were homogenized in PBS, diluted in a tenfold dilution series, and duplicate aliquots (100 µL) of the homogenate were spread on MacConkey agar (Becton, Dickinson and Company, Mississauga, ON) with 15 µg/mL chloramphenicol (Sigma Aldrich, Oakville, ON) [65]. After 24 h incubation at 37 °C, *C. rodentium* colonies were enumerated and the identity of the bacterium isolated (arbitrarily selected colonies) was confirmed with colony PCR targeting the EspB protein [89]. Primers specific for the *espB* gene, (F:5′-GCTTCTGCGAAGTCTGTCAA-3′, R:5′-CAGTAAAGCGACTTAACAGATT-3′) were used with PCR conditions that commenced with one cycle of 15 s at 95 °C, followed by 35 cycles of 45 s at 95 °C, 1 min at 57 °C, 1 min at 72 °C, and a final cycle of 5 min at 72 °C. The amplicon was 270 base pairs (bp) in size.

### Animal euthanization and intestinal sample collection

On days 14, 21 and 28 p.i., arbitrarily selected mice from each treatment were anesthetised with isoflurane and euthanized by cervical dislocation under anesthesia. To visualize the intestine and collect samples, a mid-line laparotomy was used to exteriorize the intestine [65]. A gross pathological assessment of the large intestine with photo-documentation was completed, and the length and width of the cecum and colon were measured. The cecum and colon were longitudinally incised, and luminal contents were collected and stored at −20 °C for DNA extraction and characterization of the microbiota. Sections of the distal colon were collected and immediately placed in RNAlater™ (Qiagen Inc., Toronto, ON) and subsequently stored at −20 °C for mRNA extraction. Sections from the proximal and distal colon were collected and frozen for subsequent for DNA analysis. Samples of histological examination were placed in Surgipath^®^ 10% neutral buffered formalin (Leica Biosystems, Concord, ON) for histopathologic scoring, and in Methacarn (60% methanol, 30% chloroform, 10% glacial acetic acid) for mucus analysis [90].

### Histopathologic scoring

Formalin-fixed tissues were dehydrated in increasing concentrations of ethanol (80, 95, 100%) at room temperature (RT) and placed in Histo-Clear (Diamed Lab Supplies, Mississauga, ON) prior to embedding in paraffin at 60 °C [65]. Sections (5 µm) were deparaffinized with xylene at RT, and stained with hematoxylin and eosin dyes according to a standard protocol. Tissues were scored for mucosal damage by a veterinary pathologist blinded to the treatments using an established scoring guide [26] that ranked common characteristics of mucosal damage (i.e. epithelial cell injury and hyperplasia, goblet cell depletion, crypt height, mitotic activity, and inflammation) from 0 to 4, with 4 being pronounced damage and 0 representing minimal to no damage (Additional file 1: Table S2). The maximum achievable score for tissue injury was 22.

### Visualization of mucus

To visualize mucus, colonic tissue samples were fixed overnight in Methacarn at RT [90], prior to dehydration with ethanol and Histo-Clear (Diamed Lab Supplies, Mississauga, ON). Sections (5 µm) were deparaffinized on a 60 °C heating bed, cleared with xylene, and rehydrated in a decreasing ethanol gradient (100, 90, 70, 50%) according to a standard protocol (Abcam, Toronto, ON). For mucus visualization, sections were stained with Alcian Blue (pH 2.5; American MasterTech, Lodi, CA), followed by 0.5% Periodic Acid (American MasterTech, Lodi, CA) at RT for 5 min, and Schiff’s solution (American MasterTech, Lodi, CA) at 4 °C for 15 min. Slides were examined with a Zeiss Axioskop Epifluorescent microscope (Zeiss Canada Ltd., Toronto ON).

### Short-chain fatty acid analysis

To quantify SCFAs, the cecum, proximal colon, and distal colon were collected from one mouse per treatment per replicate. The total weight of the tissue including ingesta was recorded, and after removal of the cecum, the colon was divided equally into halves as the proximal and distal colon. SCFAs concentrations were determined as described previously [65]. Briefly, sections were homogenized in PBS at a 1:9 ratio (w/v). Meta-phosphoric acid (Sigma Aldrich, Oakville, ON) was added to the homogenate at a 1:4 ratio (v/v), and incubated at RT for 30 min. Samples were centrifuged at RT for 75 min at 16,100×*g*, and the supernatants collected and stored at −20 °C. Acetate, butyrate, and propionate concentrations were quantified with a gas chromatograph (Agilent Technologies, Model 6890N with 7683 Series Injector) according to established protocols [91, 92].

### Characterization of bacterial communities in the cecum, and proximal and distal colon

Bacterial genomic DNA was extracted from distal and proximal colonic samples (mucosa-associated) using QIAGEN^®^ DNeasy Blood and Tissue Extraction kits (Qiagen Inc.). Genomic DNA was also extracted from the cecal ingesta using the QIAamp^®^ Fast DNA stool extraction kit (Qiagen Inc.); the entire cecum was removed, and all of the ingesta contents were collected for analysis. Extracted DNA was processed using an Illumina protocol for creating 16S metagenomic sequencing libraries [93]. Briefly, extracted DNA was normalized to 5 ng/µL in 10 mM Tris (pH 8.5) and 2.5 µL was PCR amplified with 5 µL of each amplicon primer specific for the V3 and V4 region of the 16S gene (F:5′TCGTCGGCA-GCGTCAGATGTGTATAAGAGACAGCCTACGG-GNGGCWGCAG-3′; R:5′GTCTCGTGGGCTCGGAGATGTGTATAAGAGACAGGACTACHVGGGTA-TCTAATCC-3′) and 12.5 µL of 2× KAPA HotStart Ready mix for a final volume of 25 µL. A PCR clean-up using AMPure XP (Beckman Coulter, Inc.) beads on a magnetic stand was performed on the 500 bp products, and an indexing PCR was used to add dual indices to each sample. Conditions included 5 µL of DNA, 5 µL of each index primer (specific non-repeating pair per sample) and 25 µL of 2× KAPA Hifi HotStart Ready mix and nuclease-free water (Qiagen Inc.) to a final volume of 50 µL per sample. A final PCR clean-up was performed on the 630 bp product. The resulting indexed DNA libraries were quantified and normalized to 4 nM, and 5 µL of each normalized library was pooled into one sample for sequencing using a MiSeq (Illumina, San Diego, CA). A PhiX control was run in parallel with the normalized DNA libraries, and both were denatured and diluted to 4 pM prior to loading onto the MiSeq cartridge.

Quantitative Insights Into Microbial Ecology (QIIME, version 1.9.0) analysis was used to assemble forward reads [94]. Barcodes were extracted from each sample and joined for each library. Libraries were then split, and sequences were filtered to include high fidelity sequences using the parameters described in [65]. These sequences (n = 1,556,473) were then chimera checked using USEARCH 6.1 software, and the resulting chimeras were filtered out prior to picking operational taxonomic units (OTU) from the Greengene reference database. In total, 1,392,105 OTUs were identified using a 97% similarity parameter, and the most common sequence was used to define the groups of similar OTUs. After, OTUs were aligned using the NAST algorithm [95] to the Ribosomal Database Project classifier of 0.8 (sequences having at least 80% similarity to reference database sequences). Taxonomy was assigned to each sequence cluster using UCLUST [96] and classified using the Greengenes reference database [97]. An OTU table was produced, and all samples were rarified so that 1,250 OTUs were randomly chosen from each sample and compared for analysis (OTU per biological sample ranged from 1,250 to 57,439). Diversity among species (β-diversity) was examined using Bray-Curtis, weighted and unweighted Uni-Frac analyses.

### Quantification of gene expression

Cytokine expression profiles were generated from total RNA that was extracted from colonic tissue using an RNeasy^®^ Mini kit (Qiagen Inc.). The concentration and quality of the total RNA extracted was analyzed using a RNA 600 Nano LabChip on an Agilent 2100 bioanalyzer. Using 1000 ng of total RNA, reverse transcription was performed using the QuantiTect^®^ Reverse Transcription Kit (Qiagen Inc. Toronto, ON). Differential expression of 15 genes involved in the Th1, Th17, and Treg immune responses were measured, as well as mucus expression, and genes involved in bacterial recognition and epithelial repair (Additional file 1: Table S3). The primers used were uniquely designed using sequence information within the NCBI database. Reference genes used to normalize measured C_t_ values were *Ppia*, *Hprt*, and *GusB* [65]. All primers were diluted to 10 nM, and high performance liquid chromatography purified. Quantitech SYBR Green Mastermix (Qiagen Inc.) was used as an indicator of double stranded DNA and product amplification. Individual PCR consisted of: 1 µL of cDNA; 3 µL of Optima water (Fisher Scientific, Edmonton, AB); 0.5 µL of 10 µM forward primer (Additional file 1: Table S3); 0.5 µL of 10 µM reverse primer (Additional file 1: Table S3); and 5 µL of SYBR green. Reactions were run in triplicate per cDNA sample (i.e. treated as observations). Quantitative PCR (qPCR) was run on an ABI 7900HT qPCR thermocycler (384-well block; Life Technologies, Burlington, ON), with an activation step of 95 °C for 15 min, and 40 cycles of 94 °C for 15 s, 58 °C for 30 s and 72 °C for 30 s, followed by 1 cycle of 95 °C for 15 s, 55 °C for 15 s and 95 °C for 15 s. The mean of the three observations was calculated, and normalized gene expression was calculated using qbasePLUS (Biogazelle, Zwijnaarde, Belgium) based on geNorm and qBase quantification models [98, 99].

### Statistical analyses

Most statistical analyses were performed using SAS (SAS Institute Inc., Cary, NC). Continuous data was checked for normality, and analysed using the MIXED procedure of SAS (SAS Institute Inc., Cary, NC). Where applicable, collection time was treated as a repeated measure; the appropriate covariance structure was utilized according to the lowest Akaike’s Information Criterion. In the event of a significant main effect, the least squares means test (LSM) was used to compare treatments within factors. Probability values ≤0.050 were considered to be statistically significant, whereas P values >0.050 and ≤0.100 were considered to be a significant trend. For bacterial community data, both SAS and Primer 7 were used to analyze data. In Primer 7, PERMANOVA and principal coordinates analyses (PCoA) were used to analyze the β-diversity, whereas, analyses of variance (MIXED procedure) with a protected LSM test were used to analyze α-diversity. Differences in the abundance of bacterial OTUs were also analyzed using analysis of variance (ANOVA) with a protected LSM test, and P values ≤0.100 were deemed statistically significant. A one-way ANOVA with a Wilcoxon two sample test for location differences, as well as the Kruskal–Wallace test was used to analyze significance between diet treatments for the histopathologic, gene expression, and LEfSe sequencing data.

## Additional file

**Additional file 1.** Supplemental tables and figures

## Abbreviations

bp: base pairs
CFU: colony-forming unit
CN: control
DF: dietary fiber
CR+: inoculated with *C. rodentium*
CR−: not inoculated with *C. rodentium*
GFP: green fluorescent protein
LA: Lysogeny Broth agar
LB: Lysogeny Broth
LRDC: Lethbridge Research and Development Centre
OTU: operational taxonomic unit
PBS: phosphate buffered saline
p.i.: post-inoculation
qPCR: quantitative PCR
RS: resistant starch
RT: room temperature
SCFA: short-chain fatty acid
WB: wheat bran
