# Supplementary material for: Impacts of resistant starch and wheat bran consumption on enteric inflammation in relation to colonic bacterial community structures and short-chain fatty acid concentrations in mice
Source: Gut Pathog. 2016 Dec 22;8:67. doi: 10.1186/s13099-016-0149-6 (PMC5178079; doi:10.1186/s13099-016-0149-6)
Supplement: Supplementary file 1 — Additional file 1. Supplemental tables and figures [file 13099_2016_149_MOESM1_ESM.pdf]

Table S1. Short-chain fatty acid concentrations (mM) in the distal colon, proximal colon and cecum of mice gavaged with PBS (white shade) and inoculated with *Citrobacter rodentium* (grey shade) 14 and 21 days post-inoculation. Mice consumed a control diet (CN) or a diet enriched with wheat bran (WB) or resistant starch (RS). * Indicates P≤0.050 when compared to CN diet, ** indicates P≤0.010 when compared to CN diet.

Diet	CN		WB		RS	
**Distal Colon**	**14**	**21**	**14**	**21**	**14**	**21**
Acetate	23.14 ± 7.86	23.46 ± 4.78	29.57 ± 15.26	39.59 ± 6.92	26.36 ± 11.35	25.54 ± 6.66
Propionate	6.23 ± 1.99	5.33 ± 1.10	4.21 ± 2.07	7.17 ± 1.42	7.94 ± 4.03	6.15 ± 2.07
Butyrate	1.55 ± 0.48	1.73 ± 0.80	**4.57 ± 2.32****	**4.60 ± 0.35****	3.40 ± 1.77	3.43 ± 1.33
Acetate	13.43 ± 8.97	10.56 ± 5.74	2.22 ±1.37	15.21 ± 4.59	5.18 ± 1.98	13.62 ± 4.98
Propionate	3.12 ± 2.29	2.68 ±1.89	0.34 ± 0.23	3.29 ± 0.56	1.04 ± 0.52	4.10 ± 1.41
Butyrate	1.26 ± 0.65	0.79 ± 0.70	0.26 ± 0.13	2.48 ± 0.76	0.42 ± 0.20	2.18 ± 0.78
**Proximal Colon**	**14**	**21**	**14**	**21**	**14**	**21**
Acetate	7.67 ± 3.86	12.56 ± 3.50	20.71 ± 9.28	14.75 ± 3.82	9.90 ± 4.95	14.85 ± 7.06
Propionate	2.25 ± 0.92	3.20 ± 1.14	3.05 ± 1.07	2.48 ± 0.46	3.20 ± 1.36	3.84 ± 1.84
Butyrate	0.44 ± 0.19	1.15 ± 0.65	**3.00 ± 2.16***	**1.93 ± 0.29***	1.86 ± 0.90	2.08 ± 0.71
Acetate	8.40 ± 4.60	12.59 ± 5.31	10.00 ± 2.58	18.50 ± 2.55	12.91 ± 3.72	5.91 ± 3.21
Propionate	2.02 ± 0.99	3.18 ± 1.43	1.58 ± 0.70	4.30 ± 1.22	2.87 ± 0.94	1.25 ± 0.72
Butyrate	1.15 ± 0.53	1.13 ± 0.58	1.12 ± 0.31	3.42 ± 1.12	1.32 ± 0.50	0.53 ± 0.30
**Caecum**	**14**	**21**	**14**	**21**	**14**	**21**
Acetate	22.46 ± 2.84	22.05 ± 3.48	31.78 ± 2.88	32.68 ± 6.54	25.71 ± 3.47	28.92 ± 3.95
Propionate	5.80 ± 0.96	5.13 ± 1.36	5.58 ± 0.74	6.01 ± 1.06	7.60 ± 1.19	7.18 ± 1.57
Butyrate	2.75 ± 1.10	2.90 ± 1.31	**6.99 ± 1.60***	**8.58 ± 2.49***	**5.93 ± 1.36***	**5.46 ± 1.20***
Acetate	29.25 ± 4.73	20.69 ± 5.68	32.83 ± 1.61	32.04 ± 5.29	25.42 ± 3.12	23.48 ± 3.41
Propionate	6.51 ± 1.28	4.86 ± 1.54	7.34 ± 0.97	7.99 ± 2.24	6.50 ± 0.94	6.78 ± 1.26
Butyrate	3.94 ± 1.03	2.35 ± 0.80	**7.80 ± 1.24***	**9.49 ± 2.14***	4.36 ± 1.12	4.31 ± 0.85

Table S2. Histological scoring parameters for mice colonic tissue. The maximum score of total inflammation is 22.

**Histological Category**	**0**	**1**	**2**	**3**	**4**
Epithelial cell wall hyperplasia	None	Mild increase, mucosal crypt columns two times the normal amount off cells, focal areas of hyperplasia	Moderate increase, mucosal crypt columns contain three times the normal amount of cells, crowding of cells in base of crypts	Marked increase, mucosal crypt columns contain four times the normal amount of cells, crowding of cells in base of crypts	Severe increase, villous distortion, mucosal crypts contain more than five times the normal amount of cells, focal dysplasia of epithelium surface
Crypt height	Normal	Mild increase, 25% increase crypt height	Moderate increase, 50% crypt height	Marked increase, 100% increase crypt height	Severe increase, >100% increase crypt height
Epithelial cell injury	None	Superficial; mild <10 surface epithelial cells shedding	Moderate, focal erosions, 11-20 surface epithelial cells shedding	Marked, multi-focal erosions of surface epithelial cells	Severe, multifocal erosions with or without deep crypt necrosis
Inflammation	None	Rare numbers of neutrophils and or mononuclear cells present within the lamina propria	Small numbers of neutrophils and or mononuclear cells present within the lamina propria	Large numbers of neutrophils and or mononuclear cells within the lamina propria that on occasion focal to multifocally extend into submucosa, muscularis and serosa	Large numbers of neutrophils and or mononuclear cells within extensive areas of the lamina propria, submucosa, muscularis and serosa
Goblet Cell Depletion	Normal	Low depletion of goblet cells, smaller size of mucin droplets	Evident depletion of goblet cells, marked decrease of the size of mucin droplets	Absent	n/a
Mitotic Activity	Normal	Mild, small increase in mitotic activity in deep crypt epithelial cells	Moderate, prominent increase in mitotic activity in the basal half of crypt epithelial cells	Marked, prominent increase in mitotic activity that extends the entire length of crypt epithelial cells	n/a

Table S3. Targets and primer sequences used to quantify the gene expression of cDNA harvested from the distal colon of mice gavaged with PBS or *Citrobacter rodentium*. *Primer sequences developed for this study.

**Primer**	**Sequence**
*Tnfα**	F:5’GATCGGTCCCCAAAGGGATG<br>R:5’GCTCCTCCACTTGGTGGTTT
*Tgfβ**	F:5’GTCCAAACTAAGGCTCGCCA<br>R:5’CATAGTAGTCCGCTTCGGGC
*RegIIIγ**	F:5’GATGCCCCATCTTCACGTAG<br>R:5’ACAAGGCATAGCAATAGGAGC
*Myd88**	F:5’ATCGCTGTTCTTGAACCCTC,<br>R:5’CCAAGTACTCGAAGCCCATC
*Muc2**	F:5’AAAGACCACAACAGGGCCAA<br>R:5’GGTCCTGGTGGTCTCCAAAG
*Il-10**	F:5’ACAGCCGGGAAGACAATAAC<br>R:5’GGCAACCCAAGTAACCCTTA
*Il-4**	F:5’AGCAACGAAGAACACCACAG<br>R:5’TCGAAAAGCCCGAAAGAGTC
*Il-17α**	F:5’GCAGCGATCATCCCTCAAAG,<br>R:5’ACGTGGAACGGTTGAGGTAG
*Il-1β**	F:5’GTGTCTTTCCCGTGGACCTT<br>R:5’GGAGCCTGTAGTGCAGTTGT
*Il-22**	F:5’TGACACTTGTGCGATCTCTGA<br>R:5’CTTGCACCGGGTGTTGACG
*Ifnγ**	F:5’ACGGCACAGTCATTGAAAGC<br>R:5’TCTGGCTCTGCAGGATTTTCA
*Tlr2**	F:5’GCTCCTGCGAACTCCTATCC,<br>R:5’CAGCAGACTCCAGACACCAG
*Tlr4**	F:5’GGCAACTTGGACCTGAGGAG<br>R:5’TTCCTTCTGCCCGGTAAGGT
*Tff3**	F:5’TCTGGCTAATGCTGTTGGTG<br>R:5’ATACATTGGCTTGGAGACAGG
*Relmβ**	F:5’TCTCAGTCGTCAAGAGCCTAA<br>R:5’AAGCACATCCAGTGACAACC
*IL-23A**	F:5’AATGTGCCCCGTATCCAGTG<br>R:5’GCAGGCTCCCCTTTGAAGAT

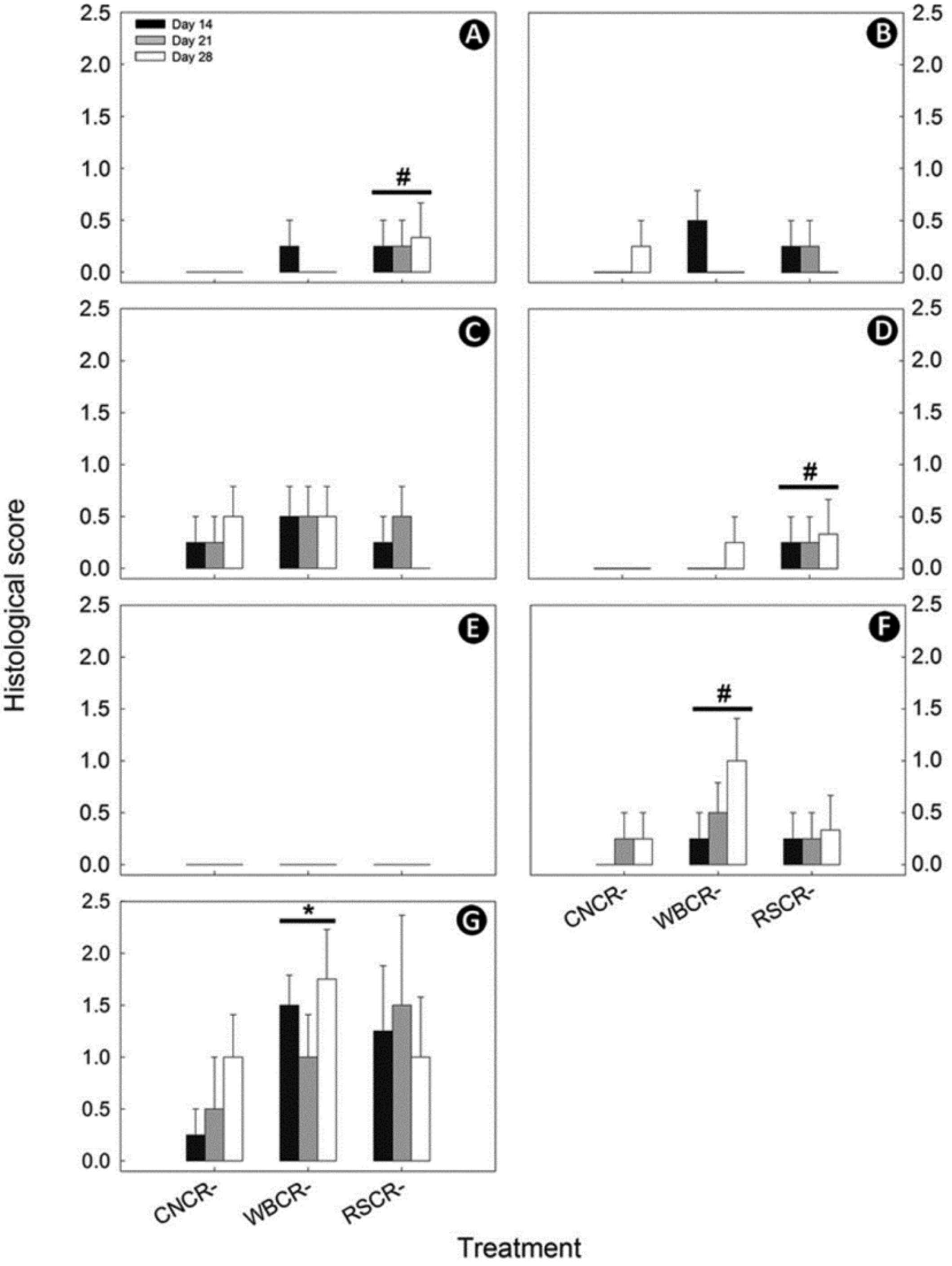

Figure S1. Histological scores measured from the proximal colon of mice gavaged with PBS (CR-) consuming a control diet (CN), or a diet enriched with wheat bran (WB) or resistant starch (RS). **(A)** Epithelial cell hyperplasia. **(B)** Crypt height. **(C)** Epithelial cell injury. **(D)** Inflammation. **(E)** Goblet cell depletion. **(F)** Mitotic acitvity. **(G)** Total average histological scores. Vertical lines associated with histogram bars represent the standard error of the mean (n=4). # Different (P≤0.100) from the CN treatment. * Different (P≤0.050) from the CN treatment.

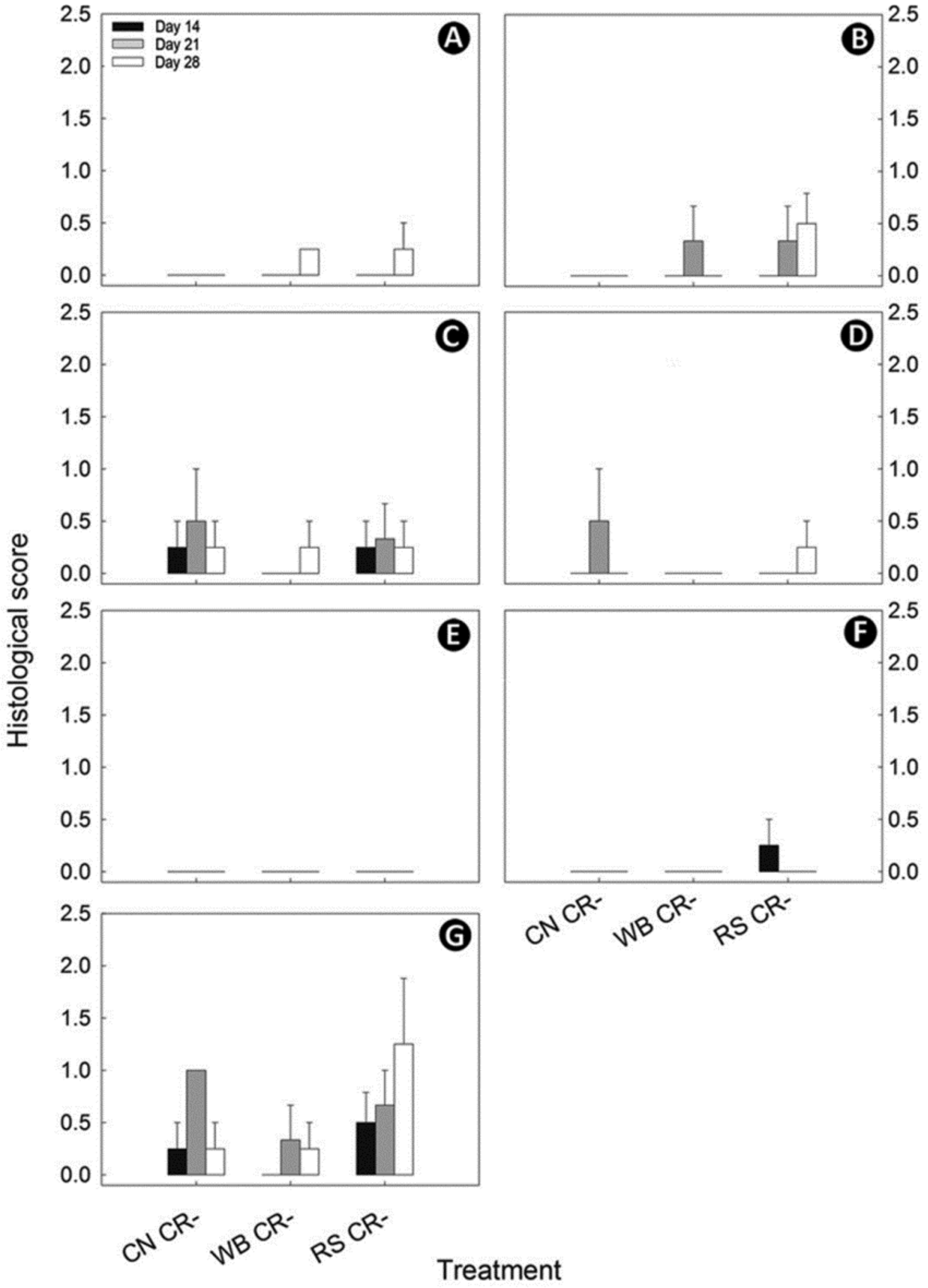

Figure S2. Histological scores measured in the distal colons of mice gavaged with PBS (CR-) consuming a control diet (CN), or diets enriched with wheat bran (WB) or resistant starch (RS). **(A)** Crypt height. **(B)** Epithelial cell hyperplasia. **(C)** Epithelial cell injury. **(D)** Inflammation. **(E)** Goblet cell depletion. **(F)** Mitotic activity. **(G)** Total average histological scores. Vertical lines associated with histogram bars represent the standard error of the mean (n=4), and no variation was measured when a vertical line is not present. There were no statistical differences among diet treatments at each of the three sample times (i.e. 14, 21, and 28 days post-inoculation).

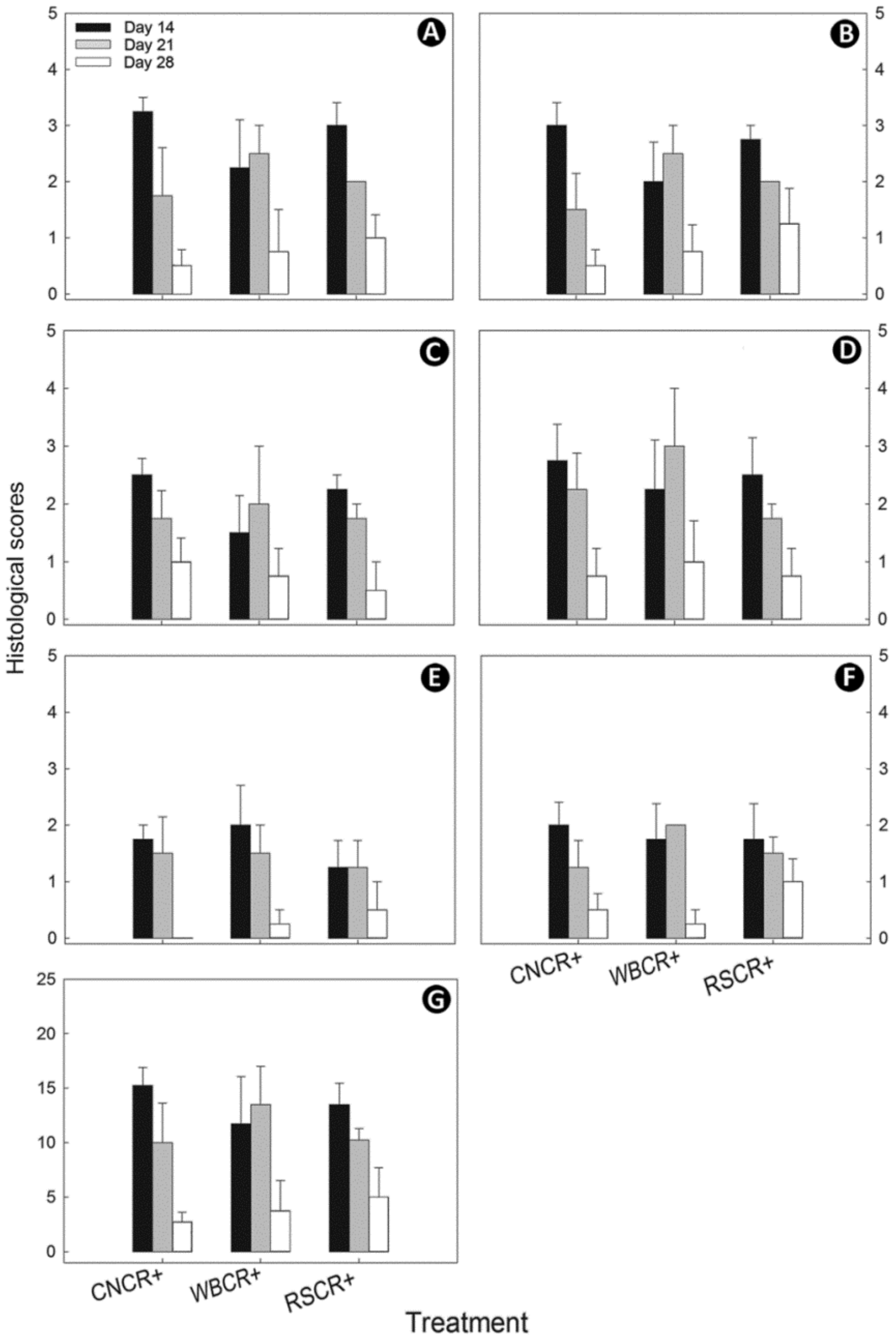

Figure S3. Histological scores measured in the distal colons of mice inoculated with *C. rodentium* (CR+) consuming a control diet (CN), or diets enriched with wheat bran (WB) or resistant starch (RS). **(A)** Crypt height. **(B)** Epithelial cell hyperplasia. **(C)** Epithelial cell injury. **(D)** Inflammation. **(E)** Goblet cell depletion. **(F)** Mitotic activity. **(G)** Total average histological scores. Vertical lines associated with histogram bars represent the standard error of the mean (n=4), and no variation was measured when a vertical line is not present. There were no statistical differences among diet treatments at each of the three sample times (i.e. 14, 21, and 28 days post-inoculation).

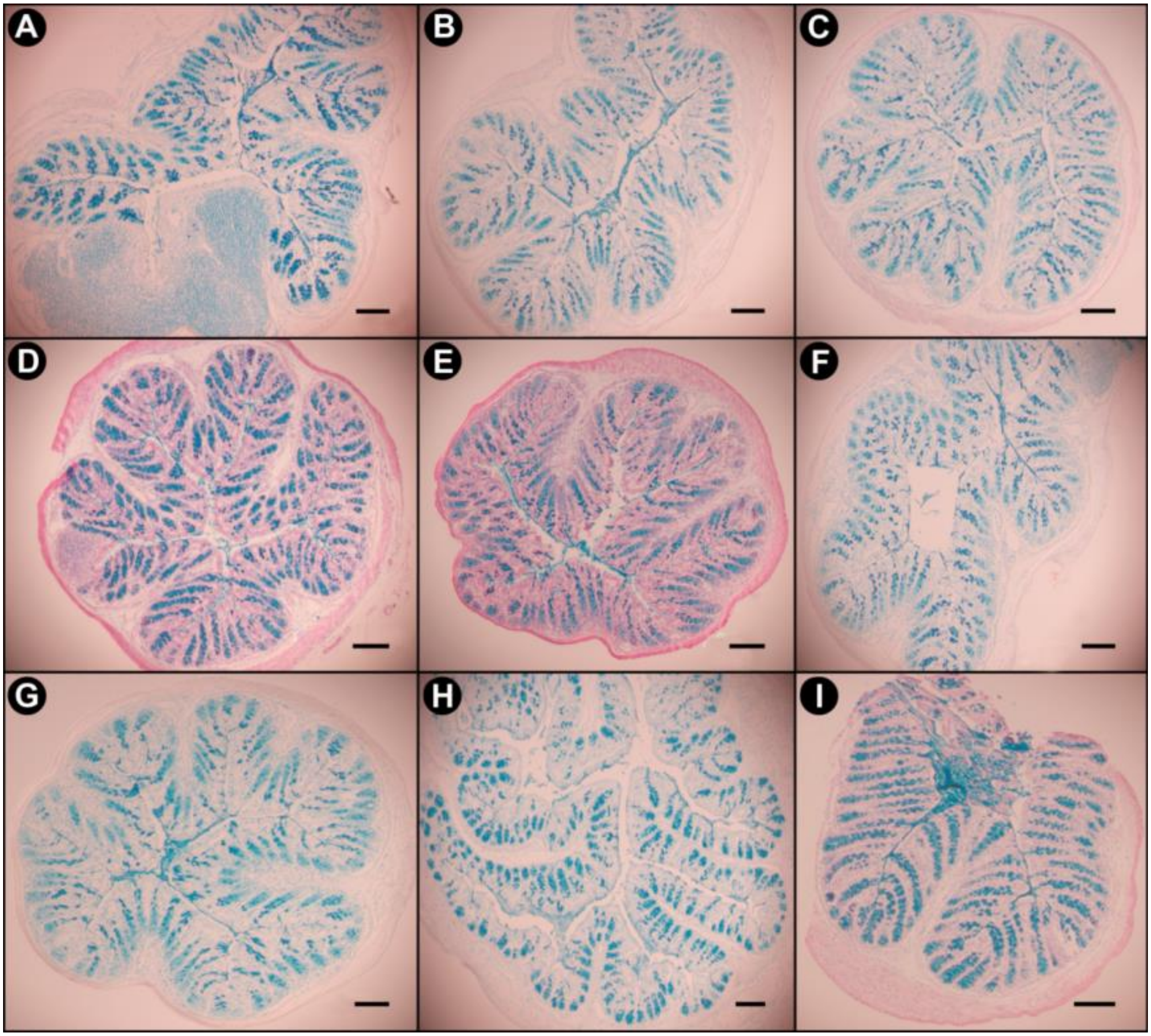

Figure S4. Mucus in the distal colons of mice gavaged with PBS (CR-) consuming a control diet (CN), or a diet enriched with wheat bran (WB) or resistant starch (RS). **(A)** CN treatment on day 14 post-inoculation (p.i.). **(B)** WB treatment on day 14 p.i. **(C)** RS treatment on day 14 p.i. **(D)** CN treatment on day 21 p.i. **(E)** WB treatment on day 21 p.i. **(F)** RS treatment on day 21 p.i. **(G)** CN treatment on day 28 p.i. **(H)** WB treatment on day 28 p.i. **(I)** RS treatment on day 28 p.i.Alcian Blue stain identifies mucus staining within the colonic sections. Bar, 100 µm.

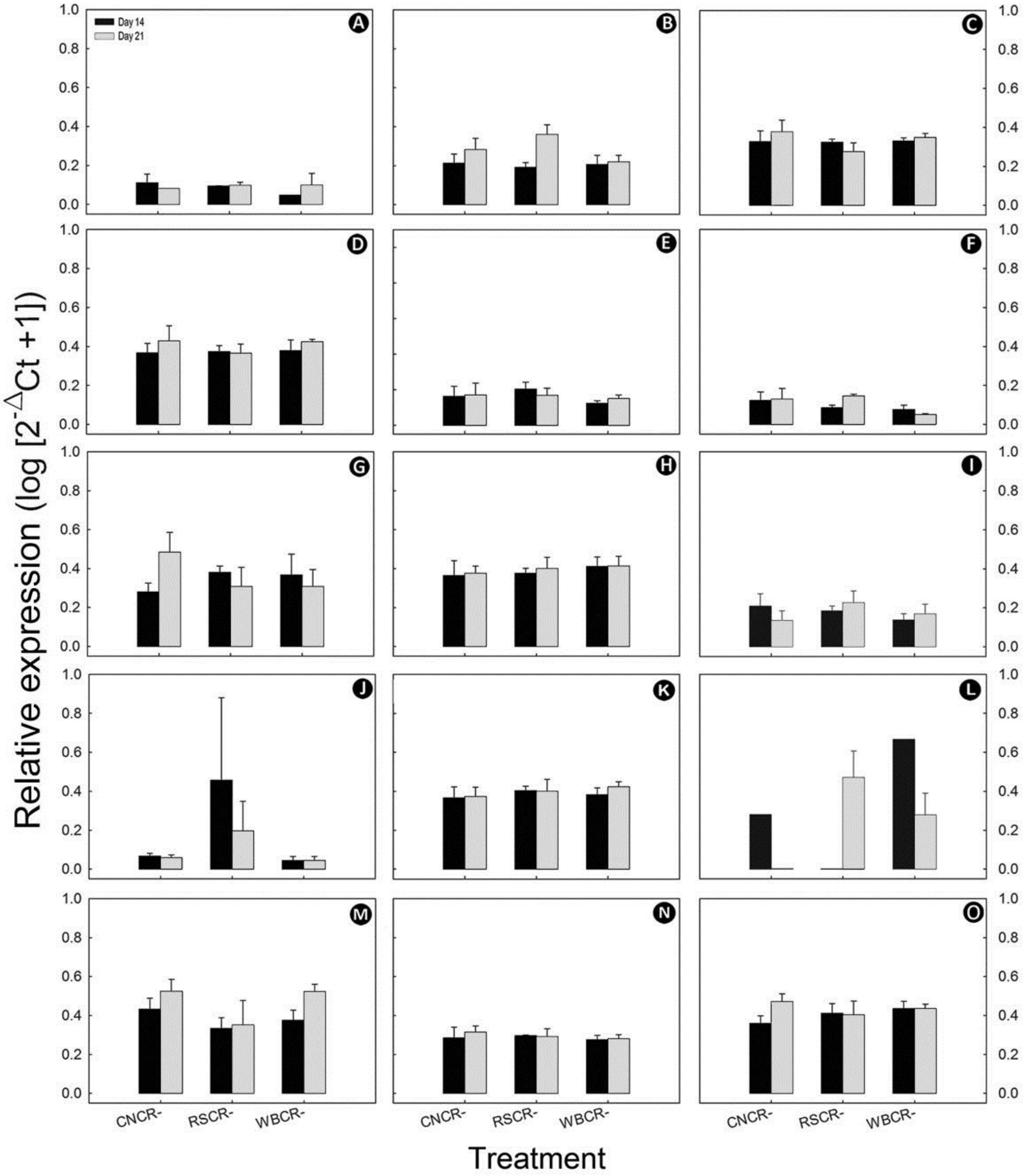

Figure S5. Expression of genes involved in Th1, Th17, Treg, and bacterial recognition and cellular repair from the distal colon of mice gavaged with phosphate buffered saline (CR-) consuming a control diet (CN), or diets enriched for wheat bran (WB) or resistant starch (RS). **(A)** *Il-17A*. **(B)** *Il-1B*. **(C)** *Tlr4*. **(D)** *Muc2*. **(E)** *Tnfα*. **(F)** *Ifnγ*. **(G)** *Il-10*. **(H)** *Tgfβ*. **(I)** *RegIIIγ*. **(J)** *Relmβ*. **(K)** M*yd88*. **(L)** *Il-22*. **(M)** *Il-23A*. **(N)** *Tlr2*. **(O)** *Tff3*. Veritcal lines associated with histogram bars represent the standard error of the mean (n=3), and no variation was measured when a vertical line is not present. There were no statistical differences among diet treatments at each of the two sample times (i.e. 14 and 21 days post-inoculation).

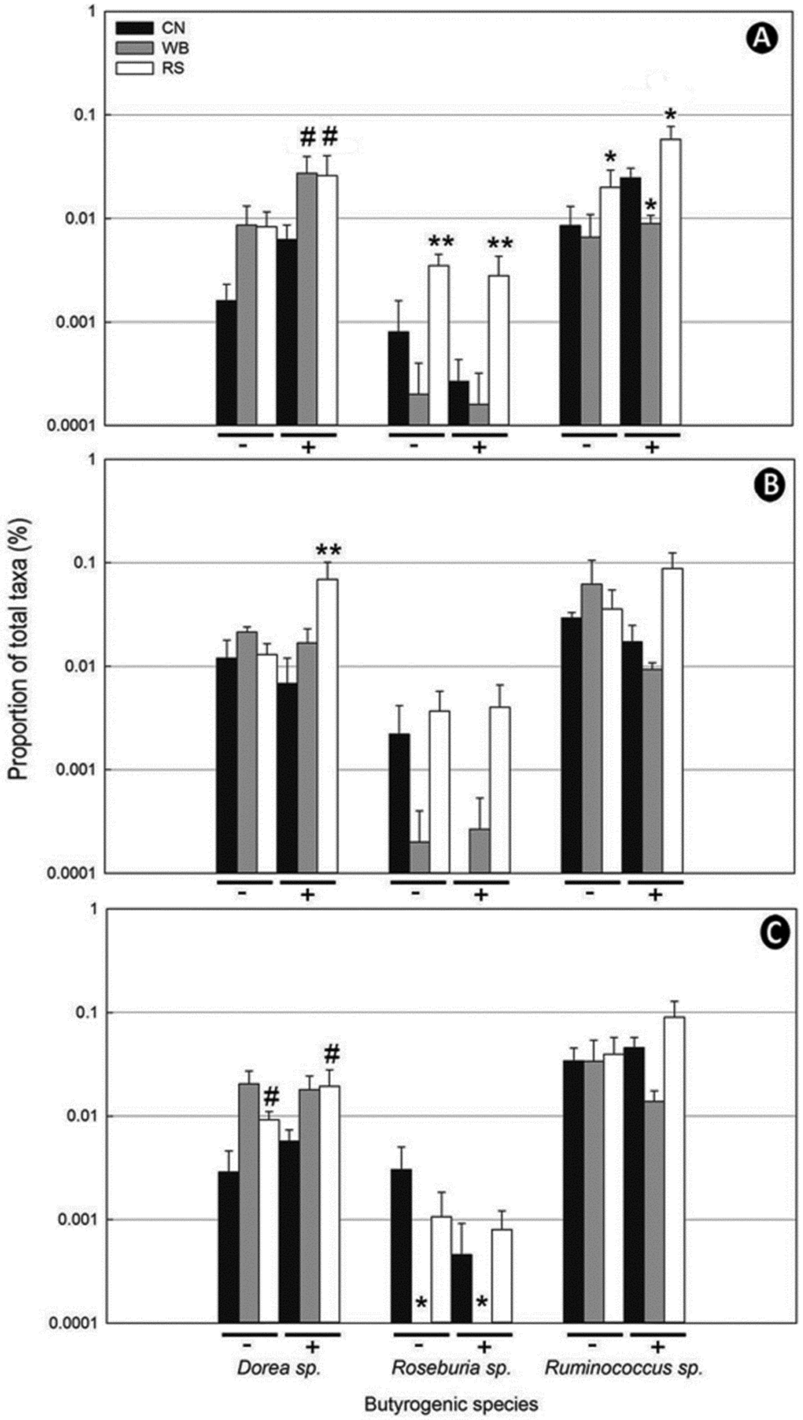

Figure S6. Proportional abundance of butyrogenic species in mice gavaged with PBS (-) and inoculated with *Citrobacter rodentium* (+) and consuming a control diet (CN), or a diet enriched for wheat bran (WB) or resistant starch (RS). **(A)** Mucosa-associated in the distal colon. **(B)** Mucosa-associated in the proximal colon. **(C) I**ngesta in the cecum. # Different (P≤0.10) relative to the CN treatment. * Different (P≤0.05) relative to the CN treatment. ** Different (P≤0.010) relative to the CN treatment.
